# *Bacteroides uniformis* CECT 7771 Modulates the Brain Reward Response to Reduce Binge Eating and Anxiety-Like Behavior in Rat

**DOI:** 10.1007/s12035-021-02462-2

**Published:** 2021-07-06

**Authors:** Ana Agustí, Isabel Campillo, Tiziano Balzano, Alfonso Benítez-Páez, Inmaculada López-Almela, Marina Romaní-Pérez, Jerónimo Forteza, Vicente Felipo, Nicole M. Avena, Yolanda Sanz

**Affiliations:** 1 Microbial Ecology, Nutrition and Health. Research Unit, Institute of Agrochemistry and Food Technology, Spanish Council for Scientific Research(IATA-CSIC), Valencia, Spain; 2 Laboratory of Neurobiology, Centro de Investigación Príncipe Felipe, Valencia, Spain; 3 Instituto Valenciano de Patología Unidad Mixta de Patología Molecular, Centro Investigación Príncipe Felipe/Universidad Católica de Valencia, Valencia, Spain; 4 Department of Neuroscience, Icahn School of Medicine, Mount Sinai, New York, NY USA

**Keywords:** *Bacteroides uniformis* CECT 7771, Food addiction, Binge eating, Reward system, Dopamine, Microbiota, Gut–brain axis, Preclinical model

## Abstract

Food addiction (FA) is characterized by behavioral and neurochemical changes linked to loss of food intake control. Gut microbiota may influence appetite and food intake via endocrine and neural routes. The gut microbiota is known to impact homeostatic energy mechanisms, but its role in regulating the reward system is less certain. We show that the administration of *Bacteroides uniformis* CECT 7771 (*B. uniformis*) in a rat FA model impacts on the brain reward response, ameliorating binge eating and decreasing anxiety-like behavior. These effects are mediated, at least in part, by changes in the levels of dopamine, serotonin, and noradrenaline in the nucleus accumbens and in the expression of dopamine D1 and D2 receptors in the prefrontal cortex and intestine. *B. uniformis* reverses the fasting-induced microbiota changes and increases the abundance of species linked to healthy metabolotypes. Our data indicate that microbiota-based interventions might help to control compulsive overeating by modulating the reward response.

**Supplementary Information:**

The online version contains supplementary material available at 10.1007/s12035-021-02462-2.

## Introduction

Food addiction (FA) has been characterized by behavioral and brain alterations leading to a loss of control of food intake [1]. This concept became popular in the second half of the twentieth century when obesity first emerged as a major public health concern. People living with obesity often have difficulties in controlling food intake, leading to overeating [2]. Indeed, obesity is frequently associated with eating disorders — for example, binge eating (BE) and addictive-like eating or FA [3–6]. The FA concept is grounded on emerging evidence that the reward mechanisms that regulate both substance abuse/addiction and overeating palatable foods are essentially the same, the activation of the reward circuitry, including the mesolimbic dopamine pathways, opioids, and cannabinoids [7–10]. However, FA differs from drug addiction in that food is essential for life and there is no chemical withdrawal syndrome [11], indeed is not officially recognized as a disease in the *Diagnostic and Statistical Manual of Mental Disorders (DSM-5)* yet [12].

Food intake control depends on both homeostatic and hedonic aspects. The main goal of the homeostatic component is meeting the energy needs to maintain body functions. In this context, the hypothalamus is one of the most important brain areas regulating dietary intake, which is mediated by the synthesis and release of orexigenic and anorexigenic neuropeptides [13]. Appetite and food intake also depend on the hedonic component, which provides pleasure. The hedonic component is regulated by central reward mechanisms that can override homeostatic satiety systems [14] and drive a self-perpetuating cycle of craving and compulsive eating that is independent of hunger and is often marked by BE [11, 15]. The main neurotransmitter associated with the reward response is dopamine, which is critical for food “wanting” [16–18]. Dopaminergic neurons project to the striatum, cortex, hypothalamus, and limbic system, including the nucleus accumbens (NAc), and regulate different physiological functions such as hormone secretion and motivated and emotional behavior [19, 20]. The reward circuitry has been researched in depth as the intake of most abused drugs causes the release of dopamine in the NAc [21–23]. Interestingly, this also occurs in animal models after consuming highly palatable foods or sucrose [24, 25].

The gut–brain axis allows bi-directional communication between the gut and the brain, mediated by hormonal, immunological, and neural signals. The gut microbiota serves as a regulator of this axis and influences the functioning of the central nervous system (CNS) and behavior, as demonstrated in intervention studies in rodents [26, 27]. For instance, the administration of *Bifidobacterium pseudocatenulatum* CECT 7765 (*B. uniformis*) was found to attenuate the obesity-associated depressive-like behavior in mice, increase serotonin concentration in the hippocampus, and modify the gut microbiota [28]. Likewise, antibiotics are known not only to reduce the total number of bacteria in the gut microbiota, but also change its composition, which could ultimately impact the physiology of the brain [29, 30]. Nonetheless, whether interventions with specific intestinal bacteria and modulation of the gut microbiota can regulate the brain reward system — and thus the food intake control — remain unclear. Although the information of the impact of the gut microbiome on behavioral response to drugs of abuse is limited, there is growing clinical and preclinical evidence of bacterial dysbiosis in subjects consuming drugs of abuse, suggesting a relationship between the two variables [31]. These studies have mainly focused on alcohol, psychostimulants, and opioid users [32–36].

In the present study, we sought to evaluate the role and mode of action of the gut microbiota in a validated rat model of FA, where loss of dietary intake control (thus triggering BE) is induced by intermittent periods of fasting and exposure to chow and palatable drink [37]. We investigated the possible control of the behavioral and neurochemical changes induced in this model by an intervention with an intestinal bacterial strain — *B. uniformis —* known to reduce body weight gain in a murine model of diet-induced obesity [38]. The model also provided clues on the possible mode of action, whereby this strain may restore the communication within the gut–brain axis and ameliorate compulsive food and drink intake and behavioral alterations brought about by FA.

## Results

### B. uniformis Reduces Caloric Intake During BE

**Fig. 1:**
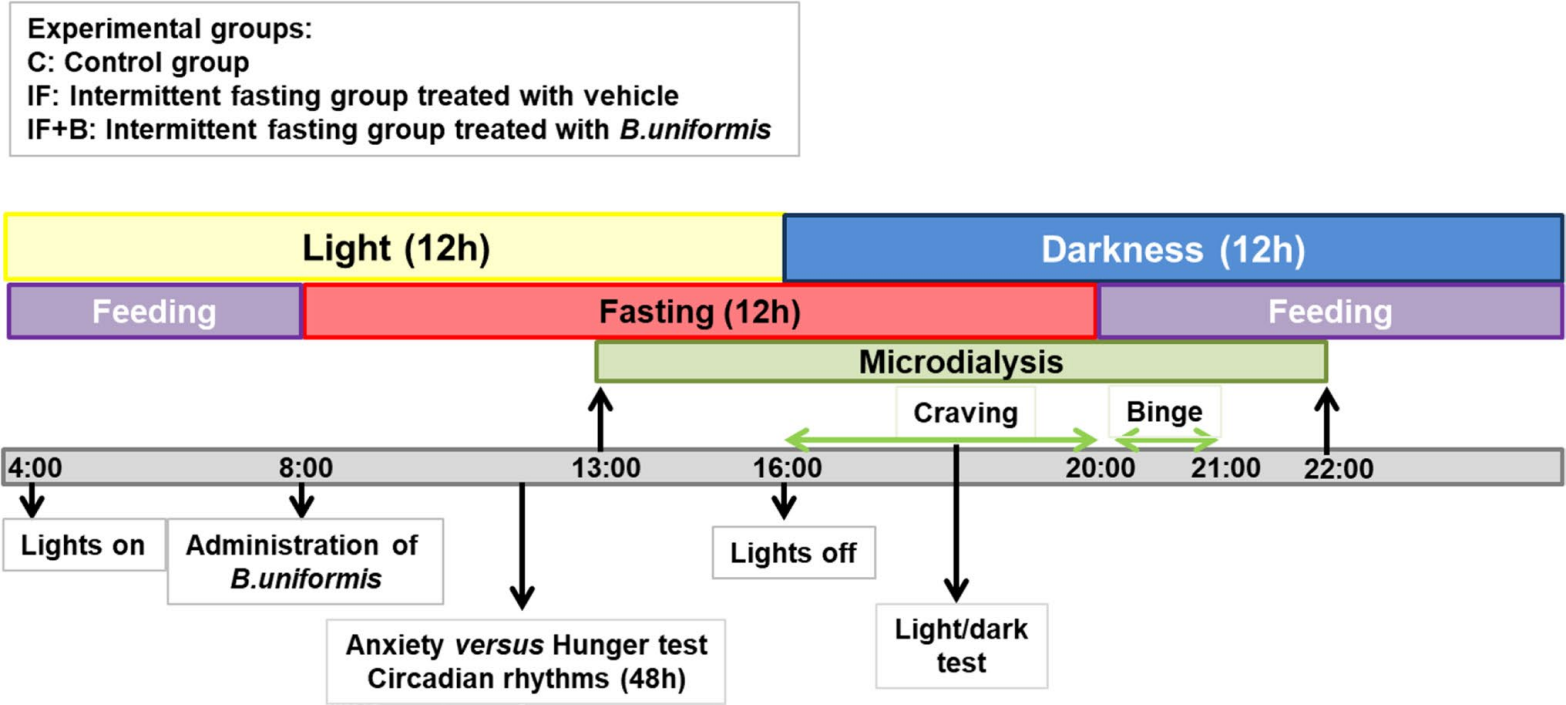
Scheme of the experimental design and assessments during the intervention in a rat FA eating model. Wistar rats were divided into three experimental groups (n = 15/each): (1) a control group (C) receiving normal diet and water during 24 h plus vehicle (10% skimmed milk), (2) an intermittent fasting (IF) group that fasts during 12 h and receives normal diet and sucrose (10%) in the drinking water during the remaining 12 h plus vehicle, and (3) an IF group treated with *B. uniformis* (IF + B) that fasts during 12 h and receives normal diet and sucrose (10%) in the drinking water during the remaining 12 h plus a daily dose of 1 × 10^8^ CFU *B. uniformis* in vehicle. The administration of the strain was at 8.00 h before fasting started. Behavioral tests were performed at different times along the study protocol. Microdialysis was performed for 11 h to collect samples at phases of the study (fasting, craving, and binge)

The FA rat model was induced by a protocol of intermittent periods of 12-h fasting — 12-h exposure to rodent chow and 10% sucrose in the drinking water. All rats also had access to plain water. Wistar rats were divided into three experimental groups: (1) a control group (C) receiving chow and water plus vehicle during 24 h, (2) an intermittent fasting (IF) group that fasted during 12 h and received chow and sucrose (10%) in the drinking water plus vehicle during the remaining 12 h, (3) an IF group treated with *B. uniformis* (IF+B) that fasted during 12 h and received chow and sucrose (10%) in the drinking water during the remaining 12 h plus a daily dose of 1 × 10^8^ colony-forming units of *B. uniformis* (scheme of the study is described in Fig. 1).

**Fig. 2:**
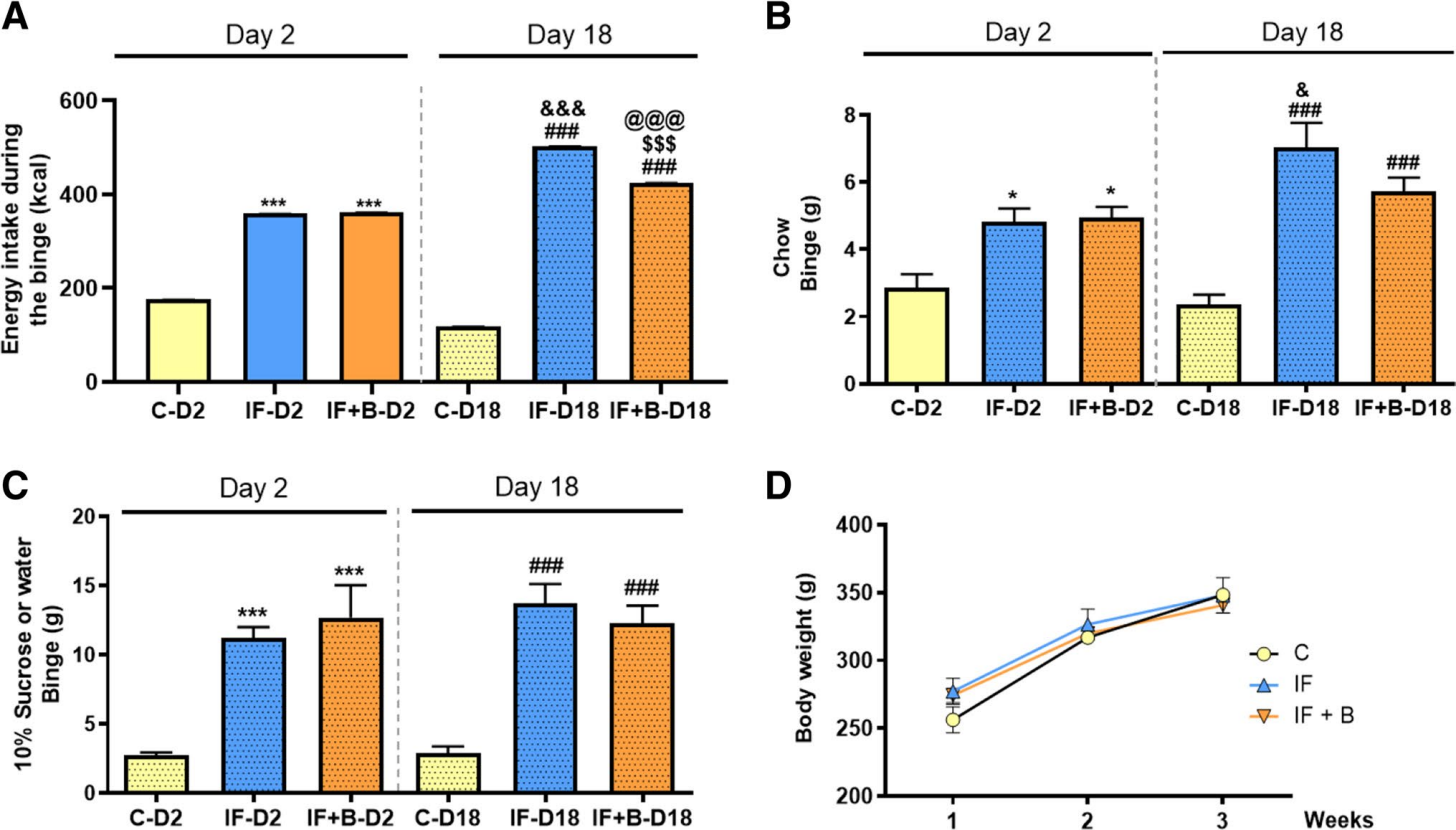
*B. uniformis* reduces total calories, chow, and sucrose solution intake during the binge with no effect on body weight gain. The sum of total caloric intake during the binge period (kcal) (**A**), mean of the chow intake (g) (**B**), mean of the 10% sucrose intake (g) (bottles were weighed before and after the binge period) (**C**), and body weight evolution (g) (**D**) in control and IF rats treated or not with *B. uniformis,* (n = 15/each). Abbreviations: C, control group; C-D2 and C-D18, control group on day 2 and 18, respectively; IF, rats that fasted 12 h daily and received vehicle; IF-D2 and IF-D18, IF group on day 2 and 18, respectively, IF + B, rats that fasted 12 h and received a daily dose of 1 × 10^8^ CFU *B. uniformis*; IF + B-D2 and IF + B-D18, IF + B rats on day 2 and 18, respectively. Rats were weighed every week during the 3-week period. One-way ANOVA followed by post hoc Tukey’s test was performed in all the figures. Statistically significant differences compared with the C-D2 group are indicated by an asterisk (*), different from C-D18 are indicated by (#), different from IF-D2 are indicated by (&), different from IF + B-D2 are indicated by ($), and different from IF-D18 are indicated by (@). *p < 0.05, ***p < 0.001, ###p < 0.001, &p < 0.05, &&&p < 0.001, $$$p < 0.001, @@@p < 0.001

The effect of the IF protocol on BE of chow and fluids (drinking water or 10% sucrose), or both, was examined at the beginning (day 2) and at the end (day 18) of the study. Analysis of the sum total caloric intake (chow and fluids) during the BE phase on the second day showed that both experimental groups (IF and IF+B) consumed nearly twice as many calories as control rats (p < 0.001) (Fig. 2A). These differences were more pronounced in both experimental groups after 18 days of a routine schedule of IF and feeding (p < 0.001) (Fig. 2A), indicating that the effect size increased along the study and validating the model. Of note, the sum total caloric intake for the IF+B group was significantly lower than for the IF group (p < 0.001) on day 18, suggesting that the addition of *B. uniformis* reduces the caloric intake during the BE phase.

Chow intake on the second day was significantly higher for the IF and IF+B groups than for the control group during the BE phase (p < 0.05) (Fig. 2B). On the last day of the intervention (day 18), the IF group ate significantly more than the control group (p < 0.001) during the BE phase, and this was significantly greater than on the second day (p < 0.05), indicating that the magnitude of the chow intake increased along the study. Likewise, the IF+B group showed an increase in chow intake compared with the control group after 18 days (p < 0.001), but these differences were not significant with respect to the second day of the experiment in within-group comparisons, suggesting that the increase in chow intake induced by the IF protocol was tempered by the administration of *B. uniformis*. Both IF groups consumed significantly more fluids (palatable sucrose solution) than the control group (water) during the BE phase on the second day (p < 0.001) (Fig. 2C). After 18 days, the IF and IF+B groups consumed significantly more fluids than the control group (p < 0.001) and slightly more than on the second day of the experiment. There were no significant differences in sucrose solution intake between the IF and IF+B groups (Fig. 2C). Examination of body weight gain along the study showed no significant changes between the 3 groups (Fig. 2D), possibly due to the short duration of the intervention.

### B. uniformis Reverses Anxiety-Like Behavior

**Fig. 3:**
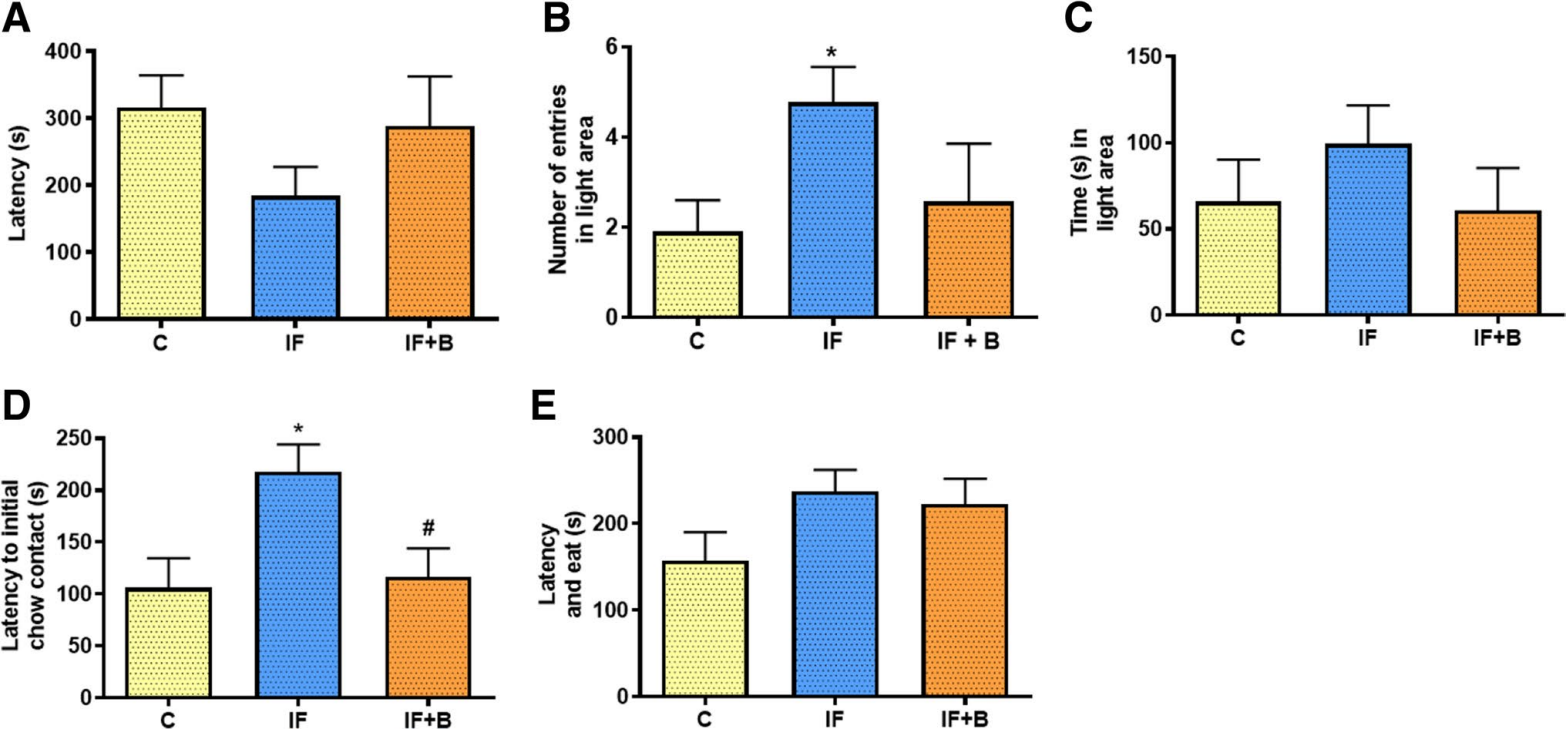
*B. uniformis* restores normal anxiety-like behavior. Light–dark box test (**A**, **B**, **C**). Latency in moving to the light area (s) (**A**), number of entries into the light area (**B**), and time spent in the light area (s) (**C**). Effect of *B. uniformis* on the anxiety *versus* hunger test (**D**, **E**). Latency to initial chow contact (s) (**D**), latency to initial chow contact, and eating (s) (**E**) (n = 15/each). Abbreviations: C, control group; IF, rats that fasted 12 h daily and received vehicle; IF + B, rats that fasted 12 h and received daily dose of 1 × 10^8^ CFU *B. uniformis*. One-way ANOVA followed by post hoc Tukey’s test was performed in **C**, **D**, and **E** and Brown–Forsythe and Welch ANOVA test post hoc Dunnett’s in **A** and **B**. Statistically significant differences compared with the control group are indicated by an asterisk (*), different from IF group are indicated by #. *p < 0.05, #p < 0.05

To question why *B. uniformis* reduces BE, we tested the possibility that this was associated with a decrease in anxiety-like behavior. Emotional factors like anxiety and stress could contribute to the dysregulation of food intake. Indeed, there is a wide literature about the relationship between anxiety and binge eating [39–42]. We thus evaluated anxiety-like behavior using two different behavioral tests: the light-dark box test and the anxiety *versus* hunger test (see Methods). The light-dark box test was performed during the craving period. Results showed that the time to move from the dark to the light area (latency) was slightly shorter in the IF group than in the control group, but this did not reach statistical significance (Fig. 3A). The IF+B group showed a trend for longer latency (hesitancy) than the IF group, with times similar to those of the control group (Fig. 3A). The number of entries into the light area, associated with increased anxiety-like behavior, was significantly higher in the IF group than in the control group (p < 0.05), whereas the IF+B group showed an intermediate number of entries (Fig. 3B). Finally, the time that rats spent in the light area showed a trend to be higher in the IF group than in the control group, whereas the IF+B group had similar values to controls. Overall, these results suggest that *B. uniformis* reverses the hunger-induced anxiety-like behavior, which could account for a better control of food intake during the binge.

The anxiety versus hunger test was performed using a white open field in the light phase. All groups had fasted for 18 h, and so the effect of fasting influenced all groups similarly. Results showed that the IF group took significantly longer than the control group to initiate the chow contact (p < 0.05) (Fig. 3D), suggesting an increase in anxiety-like behavior that overrides hunger. By contrast, the time to initiate the chow contact was significantly shorter in the IF+B group than in the IF group (p < 0.05), indicating less anxiety. Regarding the time to initiate the chow contact and eat, both the IF groups needed more time than the control group to begin eating, but this was not significant (Fig. 3E). Altogether, the results indicate that *B. uniformis* reverses the anxiety-like behavior induced by IF.

### B. uniformis Restores Normal Circadian Rhythm of Vertical Activity

**Fig. 4:**
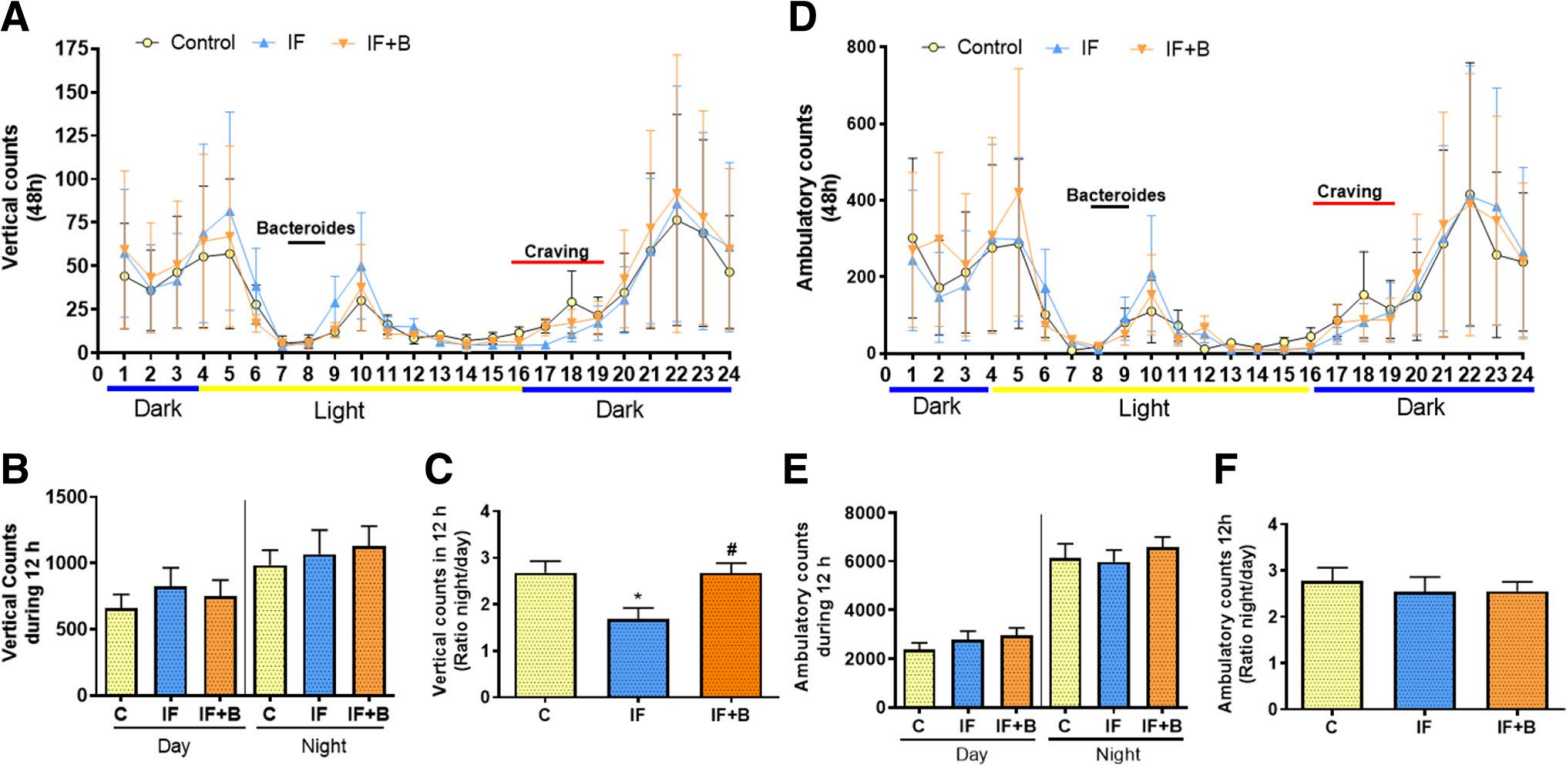
*B. uniformis* restores normal circadian rhythm of vertical activity. Circadian rhythms of locomotor activity were registered for 48 consecutive hours. Light phase comprises zeitgeber time 4.00–16.00 h and dark phase zeitgeber time 16.00–4.00 h (n = 15/each). Vertical activity every hour throughout a 24-h period is shown in **A**. **B** shows the total vertical activity during the light (day) and dark (night) phases, and **C** shows the ratio of night/day of vertical activity. Ambulatory activity every hour throughout a 24-h period is shown in **D**. **E** shows the total ambulatory activity during the light (day) and dark (night) phases, and **F** shows the ratio of night/day of ambulatory activity. Abbreviations: C, control group; IF, rats that fasted 12 h daily and received vehicle; IF + B, rats that fasted 12 h and received a daily dose of 1 × 10^8^ CFU *B. uniformis*. Two-way-ANOVA followed by post hoc Bonferroni was performed in **A** and **D** and one-way ANOVA followed by post hoc Tukey’s test was performed in **B**, **C**, **E**, and **F**. Statistically significant differences compared with the control group are indicated by an asterisk (*), different from IF group are indicated by #. *p < 0.05, #p < 0.05

We next analyzed circadian rhythms over 48 h because there is accumulating evidence that eating patterns, the microbiota, and the internal clock mechanisms are all linked [43]. We measured vertical counts (rearing) during the day and the night (Fig. 4A and B), as this could be related to hunger induced by fasting [44] or to satiety [45]. Analysis of the night-to-day ratio of vertical counts showed that this was significantly lower in the IF group than in the control group (p < 0.05) (Fig. 4C), indicating altered circadian rhythm of vertical activity. By contrast, the night-to-day ratio of vertical counts was significantly higher in the IF+B group than in the IF group (p < 0.05) and was similar to that of controls. No significant differences were found in ambulatory counts (horizontal activity) or in the night-to-day ratio of the counts (Fig. 4D–F).

### B. uniformis Does Not Modulate Hypothalamic Orexigenic and Anorexigenic Neuropeptides

**Fig. 5:**
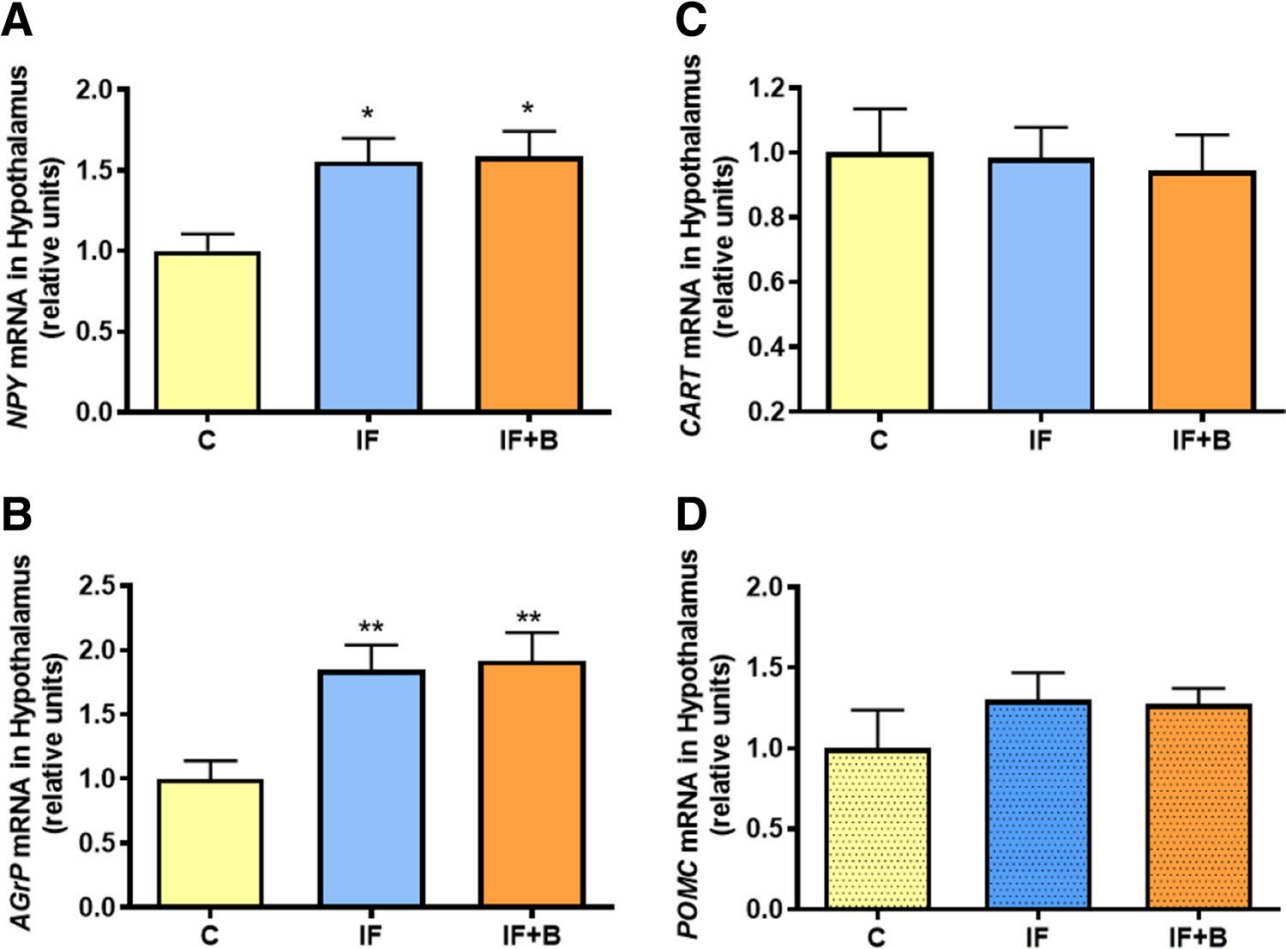
*B. uniformis* does not modulate orexigenic and anorexigenic neuropeptides. Effect of *B. uniformis* on the mRNA relative expression of neuropeptide Y (NPY) (**A**), agouti-related peptide (AgRP) (**B**), cocaine and amphetamine-regulated transcript (CART) (**C**), and proopiomelanocortin (POMC) (**D**) in the hypothalamus (n = 15/each). Abbreviations: C, control group; IF, rats that fasted 12 h daily and received vehicle; IF + B, rats that fasted 12 h and received a daily dose of 1 × 10^8^ CFU *B. uniformis*. One-way ANOVA followed by post hoc Tukey’s test was performed in all the figures. Statistically significant differences compared to the control group are indicated by asterisks (*). *p < 0.05, **p < 0.01

To elucidate whether the effect of *B. uniformis* on BE and behavior was mediated by changes in appetite/satiety signals, we evaluated the gene expression of several neuropeptides in the hypothalamus. Results showed that the expression of the orexigenic peptides neuropeptide Y (Fig. 5A) and agouti-related peptide (Fig. 5B) were significantly higher in the IF group than in the control group (p < 0.05 and p < 0.01, respectively), with similar levels in the IF+B group. Conversely, no changes were observed between groups for the levels of the anorexigenic neuropeptides cocaine- and amphetamine-regulated transcript (Fig. 5C) and proopiomelanocortin (Fig. 5D).

### B. uniformis Modulates Extracellular Neurotransmitter Levels in the Nucleus Accumbens

**Fig. 6:**
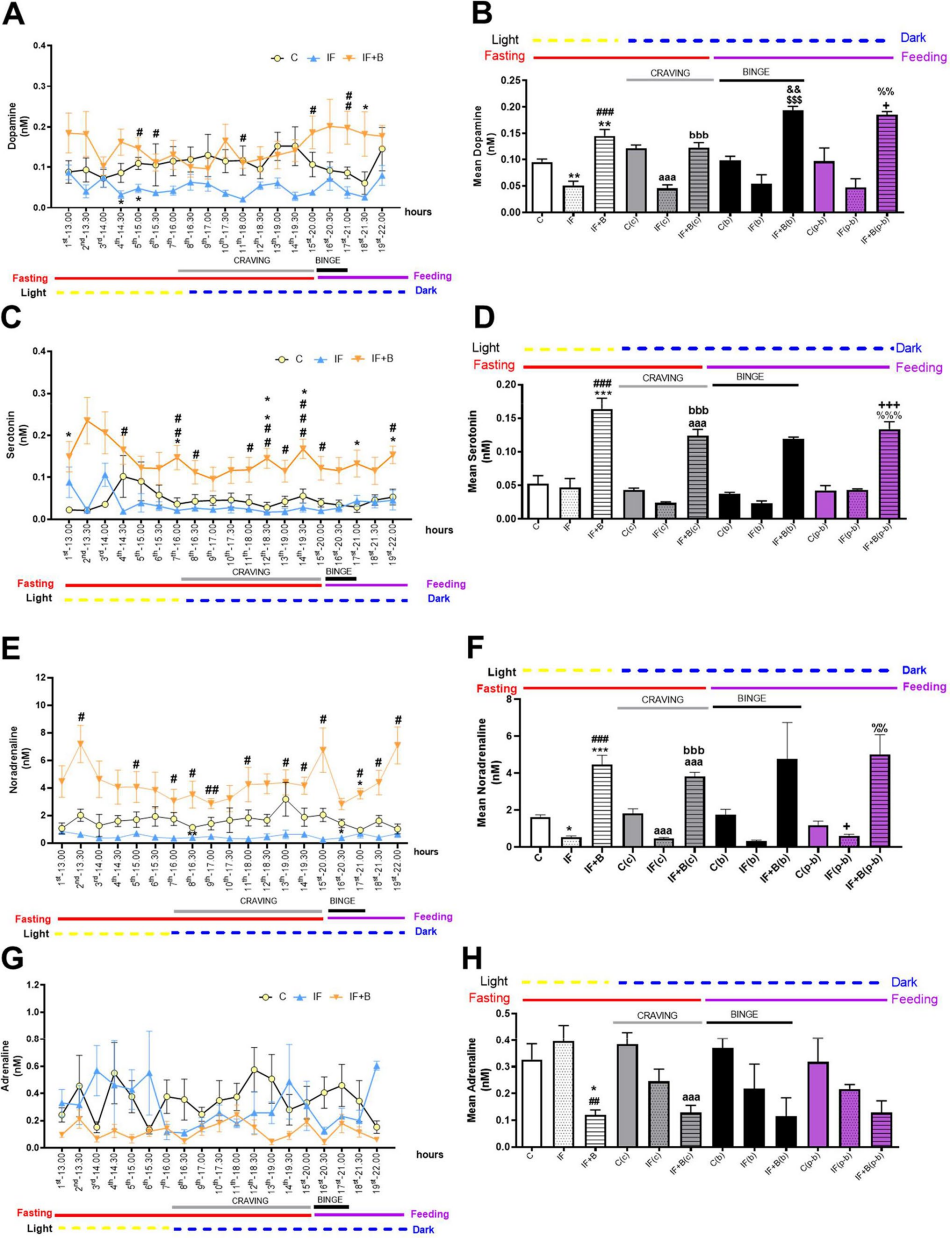
*B. uniformis* modulates neural transmitters in the nucleus accumbens as analyzed by microdialysis. Microdialysis guide was implanted in the nucleus accumbens. Extracellular concentrations of dopamine (**A**, **B**), serotonin (**C**, **D**), noradrenaline (**E**, **F**), and adrenaline (**G**, **H**) were measured by microdialysis in freely moving rats. Values are expressed in absolute value (nM) (**A**, **C**, **E**, **G**) and as the mean (nM) (**B**, **D**, **F**, **H**). Abbreviations: C, control group (n = 9); IF, rats that fasted 12 h daily and received vehicle (n = 10); IF + B, rats that fasted 12 h and received a daily dose of 1 × 10^8^ CFU *B. uniformis* (n = 9). Statistically significant differences (**A**, **C**, **E**, **G**) compared with the control group are indicated by an asterisk (*), different from IF group are indicated by #. *p < 0.05, **p < 0.01, #p < 0.05, ##p < 0.01, ###p < 0.001. Two-way-ANOVA followed by post hoc Bonferroni was performed in **A**, **C**, **E**, and **G**, and one-way ANOVA followed by post hoc Tukey’s test was performed in **B**, **D**, **F**, and **H**. Statistically significant differences (**B**, **D**, **F**, **H**) compared with the control group are indicated by an asterisk (*), different from IF group are indicated by #, different from the C(c) group are indicated by (a), different from IF(c) group are indicated by (b), different from the control(b) group are indicated by (&), different from IF(b) group are indicated by ($), different from the control(c-p) group are indicated by ( +), and different from IF(c-p) group are indicated by (%). *p < 0.05, **p < 0.01, ***p < 0.001, ##p < 0.01, ###p < 0.001, aaa p < 0.001, bbb p < 0.001, &&p < 0.01, $$$p < 0.001, + p < 0.05, +  +  + p < 0.001, %% p < 0.01, %%% p < 0.001

To determine whether the molecular mechanisms involved in the reduction of the BE were associated with the reward response, we next measured extracellular neurotransmitters (dopamine, serotonin, noradrenaline, adrenaline, and corticosterone) in the cerebral extracellular fluid by microdialysis, using a probe inserted into the NAc, while the animal was awake and moving freely (see Methods). Results showed that extracellular levels of dopamine were lower in the IF group than in controls across all 19 time points (fractions, from 13.00 to 22.00, to cover all the different phases of the study), although the differences were statistically significant only in fractions 4 and 5 (p < 0.05) (Fig. 6A). Of note, the IF+B group had significantly higher levels of dopamine than the IF group in fractions 5, 6, 11, 15 (p < 0.05), and 17 (p < 0.01), with the latter time points corresponding to the BE phase. Changes in the levels of dopamine between the IF+B and control groups were significant only for fraction 18 (p < 0.01).

We also analyzed dopamine levels by grouping the data of the microdialysis fractions that corresponded to each of the different phases of the study: light (13.00–16.00 h), craving (16.30–19.30 h), BE (20.00–21.00 h), and post-BE (21.00–22.00 h). As expected from the individual analysis, the mean concentrations of dopamine were lower in IF rats than in control rats in all study phases (Fig. 6B), although this was significant only in the light (p < 0.01) and the craving (p < 0.001) phases. The mean levels of dopamine were higher in the IF+B group than in the IF group in the light (p < 0.001), craving (p < 0.001), BE (p < 0.001), and post-BE phases (p < 0.01). Similar results were obtained when comparing the IF+B and control groups (Fig. 6B), except for the craving phase where C and IF+B groups were alike.

Analysis of serotonin levels showed that they were moderately lower in the IF group than in the control group across most of the time points (Fig. 6C). The IF+B group had higher levels of serotonin than the IF group across all time points, and this was significant in fractions 4, 8, 11, 13, 15 and 19 (p < 0.05), 7 and 12 (p < 0.01), and 14 (p < 0.001). Likewise, the IF+B group had higher levels of serotonin than the control group across all time points, and this was significant in six fractions (Fig. 6C). Analysis of the data grouped according to the study phase (Fig. 6D) showed that the IF+B group had significantly higher levels of serotonin than the IF group in the light, craving, and the post-BE phases (p < 0.001). Similarly, the IF+B group had significantly higher levels of serotonin than the control group in all the phases (Fig. 6D).

Analysis of noradrenaline levels in the CFS showed that they were generally lower in IF rats than in controls (Fig. 6E), although this was only significant in fractions 8 (p < 0.01) and 16 (p < 0.05). The IF+B group had significantly higher levels of noradrenaline than the control group in fraction 17 (p < 0.05), and, with respect to the IF group, noradrenaline levels were higher in most of the fractions: 2, 5, 7, 8, 11, 13–15, 17–19 (p < 0.05), and 9 (p < 0.01). Analysis of the grouped data (Fig. 6F) revealed that the mean noradrenaline levels were significantly lower in IF rats than in controls in the light and post-BE phases (p < 0.05) and in the craving phase (p < 0.001), whereas the mean noradrenaline levels were significantly greater in the IF+B group than in the IF group in the light and craving phases (p < 0.001) and in the post-BE phase (p < 0.01). Likewise, noradrenaline levels were significantly higher in the IF+B group than in the control group in the light (p < 0.001) and the post-BE (p < 0.01) phases.

Analysis of adrenaline in the CFS showed a trend for higher levels in IF rats than in control rats in the light phase but not in the other phases where values were generally lower (Fig. 6G). Adrenaline levels were lower in the IF+B group than in the other groups, although this was not significant (Fig. 6G). Analysis of the data grouped according to the study phase (Fig. 6H) revealed that the IF+B group had significantly lower adrenaline levels than the IF group in the light phase (p < 0.01) and significantly lower levels than the control group in the light (p < 0.05) and craving (p < 0.001) phases.

Because additional factors, including stress, can dysregulate food intake (reviewed by Sinha, 2018), we next analyzed corticosterone as a marker of the hypothalamic–pituitary–adrenal (HPA) axis. We found that *B. uniformis* also modified corticosterone levels in the NAc (Fig. S1A, B). Rats subjected to the IF protocol had lower levels of corticosterone than control rats across all time points, and this was significant for fractions 1 (p < 0.05) and 2 (p < 0.01). By contrast, IF+B rats had significantly higher mean levels of corticosterone than IF and control rats in most if not all fractions. Analysis of the grouped data (Fig. S1B) revealed that corticosterone levels were generally lower in IF rats than in control rats, but this was significant only in the light (p < 0.01) and craving (p < 0.05) phases. Conversely, corticosterone levels were significantly higher in the IF+B group than in the IF group (p < 0.001) in all the phases except the BE phase, and the same was observed when compared with the control group.

Analysis of corticosterone levels in the large intestinal content revealed a similar trend across the different experimental groups to that observed in the extracellular fluid (Fig. S1C), but the differences were not significant.

### B. uniformis Restores D1-Positive Cells in the Prefrontal Cortex and D1- and D2-Positive Cells in the Small Intestine

**Fig. 7:**
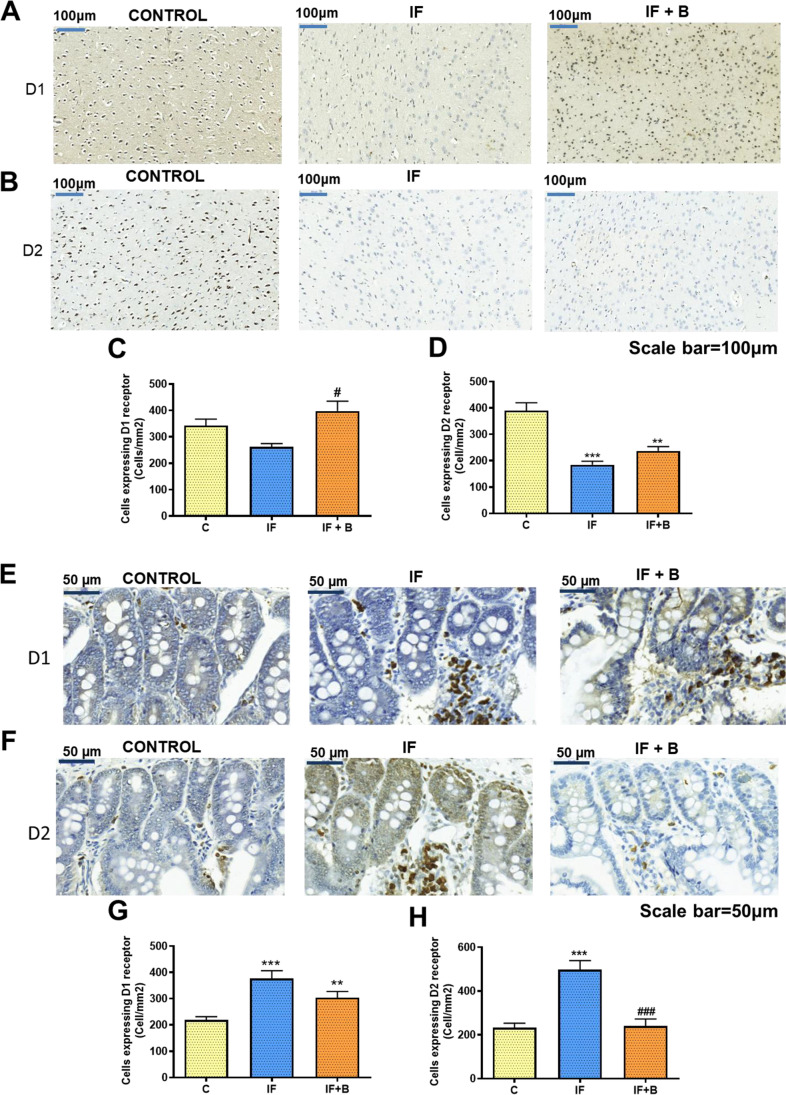
*B. uniformis* modulates D1R and D2R expression in the prefrontal cortex and the small intestine. The prefrontal cortex (PFCx) (**A**–**D**) and the small intestine (**E**–**H**). Immunohistochemistry was performed using antibodies against D1R (**A**) or D2R (**B**). Representative (× 20) magnification images are shown for PFCx and (× 56) for the small intestine. The number of D1-positive cells (**C**, **G**) and D2-positive cells (**D**, **H**) was quantified (n = 3/each). Values are the mean ± SEM of three rats per group. Abbreviations: C, control group; IF, rats that fasted 12 h daily and received vehicle; IF + B, rats that fasted 12 h and received a daily dose of 1 × 10^8^ CFU *B. uniformis*. Kruskal–Wallis test post hoc Dunn’s was test was performed in all the figures. Statistically significant differences compared with the control group are indicated by an asterisk (*) and different from IF group are indicated by (#). **p < 0.01, ***p < 0.001, #p < 0.05, ### p < 0.001. Scale bar = 100 μm for PFCx and 50 μm for the small intestine

At the end of the study, we examined the possible effects of *B. uniformis* on dopamine receptors (D1R and D2R) in the prefrontal cortex (PFCx) and in the small intestine by immunohistochemistry. Compared with the control group, the IF group showed a decrease in the number of cells expressing D1R in the PFCx (Fig. 7A and C), although this was not statistically significant. Rats administered *B. uniformis* showed normalized D1R expression in the PFCx (p < 0.05 vs IF group) (Fig. 7C). The number of D2R-positive cells (Fig. 7B and D) was significantly lower in IF rats than in control rats (p < 0.001). Rats administered *B. uniformis* showed partially normalized D2R expression in the PFCx, and the number of cells remained significantly lower than in control rats (p < 0.01) (Fig. 7D).

Analysis of dopamine receptors in the small intestinal mucosa (Fig. 7E, F) showed the opposite trend, in that the number of cells expressing D1R and D2R was significantly higher in IF rats than in control rats (p < 0.001) (Fig. 7G, H). Rats administered *B. uniformis* showed a partial restoration of the number of cells expressing D1R and a complete restoration of cells expressing D2R (Fig. 7G, H).

### B. uniformis Does Not Modulate Serotonin or Noradrenaline Receptors in the Hypothalamus

We next examine some serotonergic and noradrenergic hypothalamus receptors that could be involved in inducing satiety. We tested the expression of the serotonin receptors 5-HT1B and 5-HT2C and the noradrenaline, α -2B-adrenergic receptor in the hypothalamus, as well as the tissue levels of the corresponding neurotransmitters. Analysis of 5-HT1B expression tended to be higher in the IF group (p = 0.186) than in the control group, but this was not modified by administration of the bacterium (Fig. S2B). No differences between groups were found for 5-HT2C expression. By contrast, the levels of serotonin in the hypothalamus were significantly lower in both IF and IF+B groups than in the control group (Fig. S2C) (p < 0.001), indicating that administration of *B. uniformis* fails to restore serotonin in the FA model.

Analysis of the α -2B adrenergic receptor in the hypothalamus revealed no differences in expression between the 3 groups (Fig. S2D), and a similar result was obtained for the levels or noradrenaline (Fig. S2E).

### B. uniformis Modulates the Gut Microbiota in an FA Eating Model

**Fig. 8:**
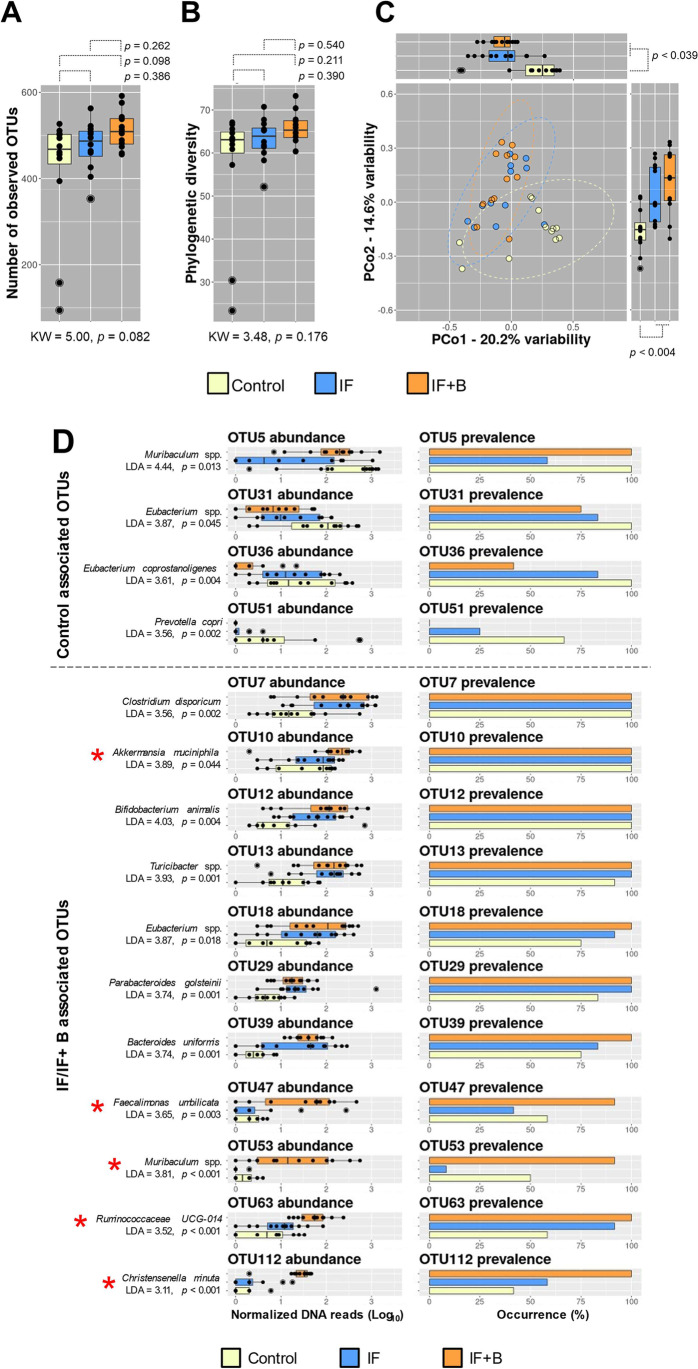
*B. uniformis* restores the gut microbiota. Alpha and beta diversity (**A**–**C**). Abundance and prevalence of OTUs (**D**). Abbreviations: C, control group; IF, rats that fasted 12 h daily and received vehicle; IF + B, rats that fasted 12 h and received a daily dose of 1 × 10^8^ CFU *B. uniformis* (n = 15/each). **A**, **B** Distribution of the total number of observed OTUs and phylogenetic diversity for all samples collected in the study. Data are presented as boxplots. P*-*values resulting from respective comparisons using non-parametric methods are shown. **C** Multivariate exploratory analysis (principal coordinate analysis, PCoA) based on the Bray–Curtis dissimilarity index between paired samples. Ellipses indicate boundaries for the confidence interval of respective distributions at 95%. Abundance and prevalence information for a total of 15 OTUs differentially present in the three experimental animal groups (**D**). Linear discriminant analysis (LDA) scoring and p-values supporting the statistical assessment are shown. The top four panels (**D**) show OTUs with higher abundances in the control group compared with the two FA groups. The remaining eleven panels show OTUs with higher abundances in the two FA groups compared with the control group. The statistically significant differences found in IF + B compared with IF group are highlighted with a red asterisk

We found no major perturbations in the α-diversity descriptors of the gut microbiota as a result of the interventions. However, we found an increase in the observed operational taxonomic units (OTUs) and the phylogenetic diversity as a consequence of the fasting procedure and the administration of *B. uniformis* compared with control animals (Fig. 8A, B). When we investigated the structure of the microbial community as a whole through multivariate analysis (β-diversity), we found a distinctive microbiota pattern that specifically differentiated the control group from the experimental groups (PERMANOVA = 2.77, p = 0.001) (Fig.8C). We thus evaluated possible differences in the abundance and prevalence of OTUs (Fig. D). When compared with the control group, the IF group showed a marked decrease in the abundance of microbial species such as *Muribaculum* spp. (linear discriminant analysis [LDA] = 4.44, p = 0.013) (the most predominant species in the murids), *Eubacterium* spp. (LDA = 3.87, p = 0.045), and *Prevotella copri* (LDA = 3.56, p = 0.002). In the IF+B group, several OTUs showed the highest prevalence or abundance compared with the other experimental group. In particular, *B. uniformis* administration increased the abundance and prevalence of *Akkermansia muciniphila* (LDA = 3.89, p = 0.044), *Christensenella minuta* (LDA = 3.11, p < 0.001), *Fecalimonas umblicata* (LDA = 3.65, p=0.003), and *Muribaculum* spp. (LDA = 3.81, p < 0.001). Of the gut microbiota alterations found in the FA model, *B. uniformis* restored the loss of *Muribaculum* species (Fig. 8, four top panels). The OTU39 identified as *B. uniformis* seemed to be more abundant in both IF and IF+B groups than in controls. Moreover, the prevalence and abundance of this bacterial species were higher in the IF+B than in the IF group, which would support the persistence of the administered bacteria in the animal intestine.

## Discussion

Our findings support the idea that the gut microbiota is involved in the loss of food control intake and especially in BE, which often occurs in people who are obese and at increased risk for mental eating disorders such as FA-like behavior [1, 46, 47]. We found that the oral administration of a commensal bacterium, *B. uniformis*, reduces caloric intake during BE episodes and restores the anxiety-like behavior that was altered by the IF protocol. Furthermore, our findings shed light on the mode of action of this bacterium, pointing to a role for *B. uniformis* in the modulation of monoaminergic neurotransmission involved in the reward mechanisms in the gut and the brain.

### B. uniformis Reduces Caloric Intake During BE by Modulating the Reward Response

We tested whether the administration of *B. uniformis* attenuates BE by modulating the reward response, focusing mainly on the dopaminergic system in the NAc, PFCx, and the small intestine. The hedonic appetite is driven by the activation of the dopamine-mediated reward pathway — the same pathway that is activated by drugs [14, 48, 49]. Our findings reveal a disruption of the dopaminergic system in rats under IF, resulting in the lower levels of extracellular dopamine in the NAc, especially during the craving phase when they were more vulnerable. Administration of *B. uniformis* corrected this in a critical phase that is often accompanied by withdrawal symptoms, which are one of the most frequent causes of relapse. We also observed a reduction in dopamine levels in the remaining phases in the IF group that could be associated with desensitization to the stimulus. Desensitization of the reward mechanism develops with repeated exposure to a particular stimulus and is characterized by disrupted dopamine signaling partly via D2R. This process reduces the satisfaction obtained with an acute stimulus that before produced pleasure, like drugs. Among other causes, desensitization is due, in part, to a lower release of dopamine in the NAc and a reduced D2R density. This process typically occurs in drug addiction; however, a similar situation seems to pertain to palatable food, which can lead to an eating disorder [2, 14, 50–52]. We observed that rats subjected to IF show low levels of extracellular dopamine but increase their chow and drink intake (stimulus) during the binge period, possibly to achieve the same level of satisfaction. We found a significant decrease in the number of D2R-expressing cells in the PFCx of IF-subjected rats with respect to controls, which is in accordance with [28] who reviewed that overeating palatable food may lead to a reduction of D2R, precipitating the compulsive eating behavior. Wang et al. [50] observed a similar reduction in D2R availability in obese individuals. D2R is represented by two isoforms: short (D2S), which is a postsynaptic receptor; and long (D2L), which is an autoreceptor [53, 54]. As we were interested in the postsynaptic response, we analyzed D2L and the postsynaptic receptor D1 in the PFCx. We focused on the PFCx because it is the brain area that governs reasoning, decision-making, and executive control [55, 56], as well as making judgments about risk and reward [57]. Accordingly, the PFCx is relevant for self-control and addictive-like eating or BE (uncontrolled food intake). Volkow et al. [58] reported a reduction in striatal D2R in obese subjects and the consequent disruptions of the orbitofrontal cortex, cingulate gyrus, and dorsolateral PFCx that could precipitate the compulsive behavior leading to overeating. We observed a non-significant decrease in the abundance of D1R-expressing cells in the PFCx of IF rats with respect to controls, which would lead to a decrease of the postsynaptic response to a stimulus. Administration of *B. uniformis* restored D1R but not in D2R expression in the PFCx. In contrast to our findings, Colantuoni et al. [59] reported that rats exposed to intermittent sugar access during 30 days showed an increase in D1R binding in the dorsal striatum, NAc core, and shell regions and a decrease in D2R binding in dorsal striatum. A possible mechanism to explain how the brain dopaminergic system is disrupted in our FA model is the following: the low extracellular levels of dopamine observed in the NAc could reflect the levels of the dopaminergic ventral tegmental area neurons that project to the NAc. The same dopaminergic neurons also project to the PFCx. Therefore, the extracellular concentration of dopamine would also be reduced in the PFCx which, in turn, might explain the reduced D2R and D1R expression in the PFCx in IF rats. Moreover, these two events (the extracellular dopamine in NAc and the dopamine receptor expression in PFCx) could independently contribute to the dysfunction in the dopamine system. The ability of *B. uniformis* to both restore D1R expression in the PFCx and increase extracellular dopamine concentration in the NAc suggests that it can partially restore signaling through the dopaminergic system in the brain, which is sufficient to mitigate the eating behavioral alterations.

We explored the local effect of *B. uniformis* in the dopamine system by analyzing D1R and D2R in the mucosa of the small intestine. The IF group showed a significant increase of both receptors in the intestine, possibly to enhance dopamine signaling, whereas the administration of *B. uniformis* moderately decreased D1R expression and totally restored D2R expression to control levels, which could contribute to normalize the signaling from the afferent vagal fibers to the brain. In fact, Egerod et al. [60] recently demonstrated in mice that D2R are expressed in the vagal afferent fibers that innervate the gastrointestinal mucosa and detect nutrients and molecules locally released in the gastrointestinal tract. Also, in a traumatic brain injury, rat model D1R and D2R were found elevated in the intestinal mucosa accompanied by intestinal mucosal barrier impairment and bacterial translocation associated with stress [61]. A cocktail of probiotics significantly improved these changes, implicating the gut microbiota in the dysregulated expression of dopamine receptors [61]. In our model, it is possible that 12 h of fasting triggers physiological stress that is reflected in the neurons of the enteric nervous system, leading to an increase in D1R and D2R expression. The mechanism by which *B. uniformis* repairs this defect could be related to its ability to modulate the gut microbiota composition and thereby the nutrient availability and signaling in the intestine or, alternatively, its ability to regulate psychological stress, as explained below.

### B. uniformis Regulates the HPA Axis Linked to the Dopaminergic System

We analyzed corticosterone as a functional marker of the HPA axis. While IF could be a source of stress, our findings reveal that the extracellular level of corticosterone in the NAc is slightly reduced in IF rats and is increased in IF+B rats. We interpret the lower levels as a reduced capacity to respond to stress (fasting) or to a decline in the alert basal state. Furthermore, corticosterone levels could be related to dopamine activity in the reward neurocircuitry. In a microdialysis study, Graf et al. [62] demonstrated that corticosterone injected intraperitoneally potentiates acute cocaine-induced increases in extracellular dopamine in the NAc and that dopaminergic neurotransmission in the NAc is necessary for corticosterone-induced potentiation of reinstatement. This could explain the association between low levels of both corticosterone and dopamine in the IF rats, which could be responsible for the increased caloric intake during the binge. Our results show that *B. uniformis* is able to modulate the HPA axis, increasing in parallel both corticosterone and other related neurotransmitters.

### B. uniformis Normalizes Anxiety-Like Behavior

We tested the hypothesis that the beneficial effect of *B. uniformis* on BE is due to its ability to reduce anxiety induced by food deprivation. We found that *B. uniformis* reverses the normal anxiety-like behavioral responses observed in the light-dark box test during the craving phase, when rats were very hungry. Several studies have reported that food deprivation alters standard behavioral patterns, increasing food seeking due to an increased need for energy [63–66]. Pierre et al. [44] suggested that food deprivation increases exploratory behavior to promote survival in rats. Our results are in line with this hypothesis as fasted rats showed an increase in the number of entries into the light area, indicating an increase in explorative and risk-taking behavior [67–70]. In addition, fasted rats also showed a trend to reduce the latency, suggesting an increase in the urgency to seek food respect to the control rats that were not hungry. Therefore, fasted rats increased the risky/exploratory behavior. Administration of *B. uniformis* decreased the exploratory behavior (the latency) in fasted rats (meaning the urgency to seek food), suggesting that it exerts an anxiolytic effect in this model. Indeed, microbiota modulation has previously been associated with improvements in mood alterations such as depression and anxiety-like behavior in animal models [71–73], including obese animal models [28]. To evaluate the anxiety-like behavior during the light phase, and under equal conditions of fasting for all the groups, we used a test based on the interplay between the motivation to consume food (manipulated by food deprivation) and the anxiety induced by exposure to the aversive open field [64, 74]. Interestingly, IF rats showed an increase in anxiety-like behavior beyond the food motivation, as they needed more time to initiate the chow contact. However, administration of *B. uniformis* reversed this, indicating an anxiolytic effect that theoretically could also contribute to reduce energy intake during the binge.

### B. uniformis Reduces the Caloric Intake But Not Through Hypothalamus Satiety Signals Associated with Neuropeptides or Neurotransmitters

We investigated whether *B. uniformis* modulates BE through the regulation of appetite/satiety signals. We found that IF induced the hypothalamic gene expression of neuropeptide Y and agouti-related peptide, which are powerful central enhancers of appetite. While this could partly explain the increased energy intake during the binge in IF rats, *B. uniformis* did not act through this pathway to alleviate BE.

We analyzed the effects of IF on serotonin and noradrenaline as an alternative mechanism through which *B. uniformis* could influence appetite/satiety signals. The IF group showed lower extracellular levels of both neurotransmitters in the NAc than the control group, which provides several explanations for the exacerbated BE. Serotonin and noradrenaline are important in both the reward response and in appetite suppression. Indeed, brain monoamines are one of the systems most frequently targeted by drugs that control energy balance — for example, sibutramine, a reuptake inhibitor of serotonin and noradrenaline and one of the most efficacious anti-obesity drugs, in spite of its adverse effects [75]. Similar to sibutramine, *B. uniformis* increased extracellular serotonin and noradrenaline in the extracellular fluid of the NAc, which could contribute to suppressing the binge. To confirm whether this was a possible mode of action of the bacterium, we analyzed serotonergic and noradrenergic hypothalamus receptors that are possibly involved in inducing satiety [76–81], but the results were inconclusive. Whereas the increase of the caloric intake observed in IF rats could be explained, in part, through hypothalamic satiety signals mediated by serotonin and noradrenaline, as the hypothalamus projects directly to the NAc [82], *B. uniformis* did not act through this pathway. This finding further supports that the major mechanism through which *B. uniformis* suppresses BE is associated with the reward response.

### B. uniformis Restores the Gut Microbiota Altered by Fasting

Finally, we examined for fasting-induced changes in the microbiota composition. We observed a slight increase in the microbiota diversity in IF rats, and this was more pronounced in the group receiving *B. uniformis*. IF has been shown to increase gut bacterial richness in animal models of multiple sclerosis [83]. In humans, increased gut microbiota diversity has been associated with healthy phenotypes [84], although there is no consensus on this issue. We observed a depletion of some species such as *Muribaculum* spp., *Eubacterium* spp., and *Prevotella copri* in IF rats, suggesting that fasting might impede the survival of some commensal bacteria. It is also possible that these microbiota changes are related to the alterations in the enteric dopaminergic system and the reward neurocircuitry in FA rats. *B. uniformis* protected IF animals against the loss of *Muribaculum* spp., supporting its ability to reverse some of microbiota alterations resulting from food withdrawal. *B. uniformis* also increased the abundance and prevalence of other bacterial taxa including *A. muciniphila, C. minuta*, *and F. umbilicata.* Of note, *A. muciniphila* is considered potentially beneficial for obesity and metabolic syndrome [85–87] and is associated with reduced hedonic eating in humans after bariatric surgery [88]. *C. minuta* is related to beneficial effects against obesity and associated metabolic disorders in humans [89]. Furthermore, genomic analysis of *F. umbilicata* reveals genes for acetate and vitamin B12 synthesis [90], which could serve as nutrients for *A. muciniphila,* as it lacks the vitamin B12 biosynthesis pathway [91, 92] and help support its growth in the ecosystem. Our results indicate that *B. uniformis* partially restores the commensal microbiota of rats subjected to IF and increases the abundance and the prevalence of some potential beneficial bacteria for host metabolism.

In summary, *B. uniformis* CECT 7771 ameliorates BE and suppresses anxiety-like behavior in a rat model of FA. We propose that the mechanisms involved in these effects are related to the ability of this strain to modulate the main neurotransmitters involved in the reward response (dopamine, serotonin, and noradrenaline) in the NAc and dopamine signaling through restoration of the expression of D1R and/or D2R in the small intestine and PFCx. *B. uniformis* also modulates corticosterone release in the NAc linked to the dopaminergic system. Lastly, *B. uniformis* CECT 7771 partially restores the fasting-induced gut microbiota alterations and increases the abundance of species associated with healthy metabolotypes. Overall, our findings support the notion that the gut microbiota plays a role in the regulation of food intake control, acting not only on energy homeostasis but also on the reward mechanisms.

Accordingly, the gut microbiota might represent a therapeutic target to improve symptoms of eating disorders linked to compulsive food consumption, such as BE or food addiction behavior.

## Material and Methods

### Methods and Protocols

#### Experimental Animal Model and Subject Details

##### Bacterial Strain and Culture Conditions

*Bacteroides uniformis* CECT 7771 (*B. uniformis*) was isolated from the stool of a breast-fed healthy infant and identified by sequencing the 16S rRNA gene, as described [93]. This bacterial strain was selected for the present study because of its ability to reduce body weight gain and metabolic alterations, possibly due to reductions in chow intake, in a murine animal model of diet-induced obesity. Bacteria were grown in Schaedler medium (broth or agar) without hemin at 37 °C under anaerobic conditions (AneroGen; Oxoid, Basingstoke, UK). Cells were harvested by centrifugation (6,000 × g for 15 min), washed twice in phosphate buffered saline solution (PBS; 130 mM sodium chloride, 10 mM sodium phosphate, pH 7.4), and re-suspended in 10% skimmed milk for animal trials. Aliquots of the suspensions were frozen in liquid nitrogen and stored at − 80 ºC until use. After thawing, live cell numbers were determined by plate counting on Schaedler agar medium after 48-h incubation and expressed as colony-forming units (CFU). For the strain tested, more than 90% cells were alive upon thawing, and no significant differences were found during storage time (2 months). One fresh aliquot was thawed every day to prevent differences in viability.

##### Animals

Experiments were carried out in strict compliance with the recommendations provided in the Guide for the Care and Use of Laboratory Animals of the University of Valencia (Central Service of Support to Research, University of Valencia, Spain), and the protocol was approved by the local ethics committee (approval number 2015/VSC/PEA/00041). Adult male Wistar Kyoto rats (170–200 g) were purchased from Charles River Laboratories (L’Arbresle Cedex, France). Rats were adapted to the facility and to a new light/dark schedule over 10 days, moving the clock forward 30 min each day. The new schedule consisted of 12 h of light (4.00 h to 16.00 h) and 12 h of darkness (16.00 h to 4.00 h). To reduce initial differences in the microbiota between the experimental groups related to the environment, we mixed the bedding used from all the cages and redistributed it in the different cages. Rats remained in these cages with the mixed bedding at least 3 days. Finally, rats were individually housed in a stainless steel cage in a temperature controlled (23 °C) room with 40–50% relative humidity. Rats were randomly divided into three groups (n = 15 rats per group) as follows: (1) a control group (C) fed a standard diet and water during 24 h plus vehicle (10% skimmed milk); (2) an intermittent fasting (IF) group that fasted during 12 h and was then fed the standard diet and sucrose (10%) in the drinking water during the remaining 12 h plus vehicle; and (3) an IF group following the same dietary pattern as in (2) but receiving *B. uniformis* (IF + B), specifically a daily dose of 1 × 10^8^ CFU in vehicle during the feeding phase (see Fig. 1). Animals were sacrificed on day 21st of the study.

### Method Details

#### Experimental Design

Figure 1 shows a schematic representation of the experimental design. Lights were turned on every day at 4.00 h for 12 h (4.00–16.00 h). At 8.00 h, 100 μl of the bacterium or vehicle (10% milk) alone was administered daily mixed in a jelly bit formulation (MediGel® Sucralose, ClearH2O, Portland, ME; comprising purified water, hydrocolloids, natural flavor, electrolyte mix, food acid, sucralose). The jelly containing bacterium or vehicle was put in the cages in a dish, and we confirmed that all rats ate the jelly. Then chow and 10% sucrose solution were removed from the cages and weighed. Only the control group had access to chow and water, whereas IF and IF + B groups only had access to water. All groups had continuous access to plain water. Later, at 16.00 h, the lights were turned off. At that moment, the craving (16.00–20.00 h) started. The craving produces the urge to consume a drug, in this case chow and especially 10% sucrose. The craving was precipitated because rats fasted during 8 h in the light phase (8.00–16.00 h, the “inactive phase” because rats are naturally nocturnal). When the lights were turned off (16.00 h), the rats started their “active phase” without access to chow or 10% sucrose for 4 h more, and, consequently, rats started to experience withdrawal symptoms. Thus, IF and IF + B groups fasted during 12 h (8 h during the light “inactive” phase plus 4 h during the dark “active” phase). At 20.00 h, all the groups had access to the chow and water, and IF and IF + B group also had access to 10% sucrose solution during the following 12 h. The first hour of the 12-h period was termed the “binge”, during which we measured how much they ate and drank. Then, rats had free access to chow and drink until 8.00 h.

### Behavioral Tests

#### Binge Eating

Binge eating was measured by weighing the individual chow and the bottles before and after the binge (1 h) in each cage every day.

#### Light–Dark Box Test

This test is one of the most widely used tests to measure anxiety-like behavior in mice or rats and is based on a conflict between the innate aversion to brightly illuminated areas and the spontaneous exploratory behavior in a novel environment [94]. The test was performed on day 12th of the experiment. It consists of a light–dark Plexiglas box (44 × 44 × 40 cm). The dark compartment is one-third of the box with black walls, and two-thirds is the light compartment with white walls. The box is illuminated by 300 lx light, and an open door (10 × 7.5 cm) communicates the light and dark compartments. During the dark phase, rats were individually placed in the dark box (20 lx) and allowed to freely explore the whole box (light and dark) for 10 min, which was recorded by a video camera (Sony EXview HADCCDII, Cornellá, Barcelona, Spain). The latency to emerge from the dark side of the chamber (four paws in the lighted side), the visits to the light compartment and the time spent there are all measures of anxiety [95]. All the parameters were measured and analyzed comparatively (Smart Video Tracking Software, Panlab, Barcelona, Spain). The test was performed during the craving in dark phase.

#### Anxiety Versus Hunger Test

This test measures rat anxiety against the motivation of hunger in an open field test, which is based on the conflict between neophobia/anxiety and hunger [64]. Before the test, all rats were fasted for 18 h with ad libitum access to water, and, therefore, the animals could not totally follow the daily schedule of fasting and refeeding in this particular case. The test was performed on day 14th of the study. The day before, rats receive the oral administration of *B. uniformis* at 8:00 h, but instead of being submitted to fasting, the rats had access to food and water until 18:00 h. Then the rats were fasted, and the fasting time lasted from 18:00 h to 12:00 h noon the following morning with ad libitum access to water (18 h). The test was performed at 12:00 h and finished before the darkness phase. During the light phase, rats were individually placed in one corner of a white open field (45 × 90 × 60 cm) facing the center where a Petri dish (8-cm diameter) of gelatine (the same as that used to administer *Bacteroides uniformis* CECT 7771 (*B. uniformis*) was available. The open field was illuminated by 400 lx light (bright laboratory light). Rats were observed and recorded (Sony EXview HADCCDII) for 8 min, and their behavior was analyzed for the following parameters: latency to initial chow contact (s), latency to initial chow contact and eating (s), and incidence of chow intake (% of rats per group). We corroborated that the rats ate by weighing the Petri dish before and after each test. At the end of the experiment, animals were returned to their home cages and to ad libitum chow from 16:00 h to 8:00 h of the following morning. All parameters were analyzed comparatively with Smart Video Tracking Software. The open field was cleaned with 5% ethanol after each test, and the Petri dish was filled with new gelatin for each animal.

#### Circadian Rhythms of Spontaneous Motor Activity

Motor activity was measured using an actimeter (Med Associates, St. Albans, VT). The actimeter consists of a chamber (43 × 43 × 31 cm) composed by a 2-dimensional (X and Y axes) square frame, a frame support, and a control unit. Each frame has 16 × 16 infrared beams for optimal subject detection. The infrared photocell system allows the evaluation and recording of general activity, locomotor, and stereotyped movements or rearings or exploration continuously during 48 h in 12:12 h light/day cycle conditions and following the same schedule of fasting and feeding with chow and 10% sucrose. The motor activity was measured between days 15th to 18th and started at 12:00 noon in the morning. Data was recorded at intervals of 5 min. The software associated with the actimeter (Med Associates) gave information about the ambulatory counts and vertical activity (rearing). Light onset was at 4.00 h, and the start of the dark period (16.00 h) was defined as a zeitgeber time (ZT) 12.

### In Vivo* Brain Microdialysis in the NAc*

Rats were anesthetized with isoflurane, and a microdialysis guide (MD-2251, Omega-ring Intracerebral Guide Cannula and Stylet, BASi) was placed stereotaxically in the posterior medial accumbens shell (AP, + 1.2 mm; ML, 0.8 mm; and DV, 4.0 mm) with reference to bregma and the surface of the level of the skull. At 48 h after surgery, each rat was placed in the microdialysis chamber, and a probe (MD-2200, Brain Microdialysis Probes) was inserted in the freely moving rat. Probes were perfused (3 μL/min) with artificial cerebrospinal fluid (145 mM NaCl; 3.0 mM KCl; and 2.26 mM CaCl2 in 2 mM phosphate buffer at pH 7.4). After a stabilization period of at least 2 h, samples were collected every 30 min and quickly frozen in liquid nitrogen and kept at − 80ºC for analysis by UPLC-MS/MS. In total, 19 samples of 30 min (3 μL/min) were collected per rat. The microdialysis was performed from day 17th to 19th of the study.

### Neurotransmitter and Corticosterone Analysis by UPLC-MS/MS Spectrometry

Chromatographic separation and assessment of analytes (dopamine, serotonin, noradrenaline, adrenaline, and corticosterone) were performed using a Waters® Acquity® TQD tandem quadrupole UPLC/MS/MS system using similar protocols to those used in González et al. [96]. Analytes (5 µL injection volume) were separated on a Kinetex C8 column (1.7 µm, 2.1 × 100 mm, Phenomenex, Madrid, Spain). Mobile phase A (0.5% formic acid in water [v/v]) and mobile phase B (% acetonitrile) were eluted at a flow rate of 0.35 mL/min according to the following gradient: 100% A (0–2.5 min), 100% A (2.5–4.4 min), 35% A and 65% B (4.4–6 min), 35% A and 65% B (6–6.1 min), and 100% A (6.1–10 min).

Mass spectrometry conditions for the detection of the analytes were as follows: capillary, 3.5 kV; extractor, 5 V; RF lens, 0.3 V; source temperature, 120 ºC; and desolvation temperature, 300 ºC. MS1 parameters were LM resolution, 13; HM resolution, 13; and ion energy, 1. MS2 parameters were LM resolution, 13; HM resolution, 13; ion energy, 0.7; multiplier 650 V; gas flow desolvation of 1100 L/h; and cone, 100 L/h. Spectra were acquired in positive ionization multiple reaction monitoring (MRM) mode with an interchannel delay of 0.07 s. The MRM transitions monitored were m/z 154.1 → 137.1 for dopamine, 177 → 132.2/160.03 for serotonin, 170 → 134.9/152 for noradrenaline, 184.3 → 166.1 for adrenaline, and 347 → 97.1/121.1 for corticosterone.

For calibrations, stock solutions of analytes (1 mg/mL) were freshly prepared in acetonitrile–water (10:90, v/v) and stored at − 80 ºC. Working solutions of 0.1,1,10,100, and 1000 ng/mL were freshly prepared in the same solvent for each run. The area under the peak of the analytes was used as a function to quantify the respective concentrations.

### Neurotransmitter Analysis by HPLC

Noradrenaline and serotonin were measured in dissected tissue from the hypothalamus using high-pressure liquid chromatography (HPLC). Samples were frozen in liquid nitrogen and stored at − 80 °C. Samples were thawed and 200 μL of trifluoroacetic acid were added per 100 mg of tissue. Samples were homogenized using a Tissue Micro pestle (Labbox, Vilassar de dalt, Barcelona, Spain), centrifuged at 10,000 × g for 15 min at 4 °C, and supernatants were filtered through 0.45-μm filters (Millipore) and collected. Chromatographic separation was performed on an Agilent 1220 infinity series HPLC system (Agilent, Waldbronn, Germany), equipped with a degasser, a binary pump, and an auto-sampler. Tissue homogenates were analyzed using a Poroshell 120 EC-C18 column (4.6 × 50 mm, 2.7 μm i.d.). The mobile phase comprised (A) 0.1% formic acid in deionized water (18 MΩcm) at pH = 2.2 and (B) 0.1% formic acid in deionized water/acetonitrile (20:80, v/v). The following gradient program was used: 0–9 min, isocratic period at 100% (A); 9–14 min, linear gradient to 75% (B); 14.1–15 min, isocratic period at 100% (B); and 15–18 min, isocratic period at 100% (A). A 5 min pre-equilibration period was used between each run. The flow rate was 0.8 mL/min; the column temperature was 25 °C; the detector was set at 254 nm wavelength; and the injection volume was 10 μl. Neurotransmitters were identified by their retention times as determined by using commercially available standards (Sigma-Aldrich. St Louis, MO). Results are expressed as nanograms of neurotransmitter per gram of fresh tissue weight.

### Immunohistochemistry

Three animals per group were deeply anesthetized with sodium pentobarbital and transcardially perfused with 150 mL of 0.9% saline followed by 250 mL of 4% paraformaldehyde in 0.1 M phosphate buffer (pH 7.4). The brain and small intestine were removed and post-fixed for 24 h at 4 ºC in the same fixative solution. Five-micrometer-thick paraffin sections were cut and mounted on coated slide glass. Sections were processed with the EnVision Flex + Kit (DAKO) to block endogenous peroxidase activity for 5 min and then incubated with an anti-dopamine receptor D1 (Abcam, ab40653) or anti-dopamine D2L receptor (Abcam, 191,041) antibody at a dilution of 1:500 for 40 min. The reaction was visualized by incubation with Envision Flex + horseradish peroxidase for 20 min and, finally, with diaminobenzidine for 10 min. Sections were counterstained with Mayer’s hematoxylin for 5 min. D1R- and D2R-positive cells were manually counted with ImageJ by two blinded investigators, and the results (the mean of the two blinded counts) were expressed as cells/mm^2^. At least 9 different sections were counted for each rat. Threshold and analyzed particle functions, the intensity thresholds, and size filter were applied. The results were converted from pixels to micrometers. For the immunohistochemistry analysis, we used at least 9 different sections for each rat. Sections were quantified using ImageJ. Cells were counted using different magnifications: (× 20) prefrontal cortex and (× 56) small intestine. Results represent the mean of the number of cells per area (mm^2^).

### Corticosterone Extraction from Intestinal Content

Intestinal content was stored at − 80 °C until processing. Approximately 100 mg of intestinal content was dried in an evaporator for 2 h (Concentrator plus/Vacufuge plus, Eppendorf, Germany). Then, 2.5 ml of 100% ethanol was added, and the mixture was vortexed and heated for 20 min in a bath at 30 ºC. Tubes were centrifuged for 15 min at 5000 rpm and the supernatant (2.5 mL) was placed into a 15-mL tube. Then, 1.25 mL of ethanol was added to the sample, which was vortexed and centrifuged again for 15 min at 5000 rpm. The supernatant was collected and added to the previous supernatant (2.5 mL), and the 5 mL was evaporated and reconstituted in 1 mL of methanol and frozen at − 80 ºC. Samples were analyzed by UPLC-MS/MS spectrometry.

### RNA Isolation and RT-qPCR Analysis

The hypothalamus was immediately snap-frozen in liquid nitrogen, and RNA was extracted using the TRIsure Bioline reagent (Bioline, London, UK) with a homogenization step using a UP400S ultrasonic processor (Hielscher, Teltow, Germany). RNA quality was assessed by measuring the absorbance ratio (260/280) on a NanoDrop ND-1000 spectrophotometer (Thermo Scientific, Wilmington, DE). RNA (2 μg) was subjected to reverse transcription (Applied Biosystems, Foster City, CA), and 20 ng of the resulting complementary DNA (cDNA) was used as a template for real-time PCR amplification. Messenger RNA (mRNA) levels for specific genes were determined on a LightCycler 480 instrument (Roche, Branchburg, NJ) using LightCycler 480 SYBR Green I Master chemistry with the primer pairs for the corresponding gene. Β2-microglobulin (β2M) was used as a housekeeping gene. The sequence and information for the primers are shown in Supplemental Table 1. All amplification reactions were performed in triplicate, and average threshold cycle (Ct) numbers of the triplicates were used to calculate the relative mRNA expression of candidate genes. The magnitude of change of mRNA expression for candidate genes was calculated using the standard 2^−hΔΔCt^ method. All data were normalized to the housekeeping gene and expressed as percentage of control.

### DNA Extraction and Sequencing

Approximately 150 mg of intestinal content of the cecum was processed for DNA isolation using the MoBio PowerSoil™ Kit with prior steps for improving cell lysis. Briefly, chemical cell lysis was performed by incubating samples with 300 μL PBS containing 500  g lysozyme and 10 U mutanolysin at 37 ºC for 1 h. This was followed by a mechanical cell disruption step in a Mini-Bead Beater apparatus (BioSpec Products, Bartlesville, OK) with two cycles of shaking during 1 min and incubation on ice between cycles. Genomic DNA was quantified by spectrophotometry (NanoDrop). The V3-V4 hypervariable regions from bacterial 16S rRNA gene were amplified in triplicate by PCR using 20 ng DNA in 25 PCR cycles at 95 ºC for 20 s, 40 ºC for 30 s, and 72 ºC for 20 s. Samples were tagged with barcodes to allow multiplexing during the sequencing process. Phusion High-Fidelity Taq Polymerase (Thermo Scientific) and the barcoded primers S-D-Bact-0341-b-S-17 (CCTACGGGNGGCWGCAG) and S-D-Bact-0785-a-A-21(GACTACHVGGGTATCTAATCC), which target a wide range of bacterial 16S rRNA genes [97], were used during the PCR. Dual barcoded PCR products, consisting of ~ 500 bp, were purified from triplicate reactions with the Illustra GFX PCR DNA and Gel Band Purification Kit (GE Healthcare, Little Chalfont, UK) and quantified by Qubit 3.0 and the Qubit dsDNA HS Assay Kit (Thermo Fisher Scientific). Samples were multiplexed in one sequencing run by combining equimolar quantities of amplicon DNA (~ 50 ng per sample) and sequenced in one lane of the Illumina MiSeq platform with 2 × 300 PE configuration (CNAG, Barcelona, Spain).

## Quantification and Statistical Analysis

### Analysis of Behavior Test

#### Analysis of Circadian Rhythms of Spontaneous Motor Activity

The analysis of the data was performed using the software associated with the actimeter (Med Associates, St. Albans, VT). Data were recorded at intervals of 5 min; therefore, each hour recorded 12 sets of data, which was used to calculate the mean of each hour per parameter and animal. We recorded the data over 48 h; hence, we had a mean of 2 for each hour and animal. We represented the mean of each hour per group (Figs. 4 A, D). Then, we organized the data (mean of each hour) from 1 to 24 h and we summarized the light hours (12 h) and the dark hours (12 h) for each rat. These data are represented in Fig. 4B, C, E, and F.

### Bioinformatics for Microbiota Analysis

For bioinformatic analysis, raw data were delivered in *fastq* files, and pair ends with quality filtering were assembled using Flash software [98]. Sample de-multiplexing was carried out using sequence information from the respective DNA barcodes and *MOTHUR* v1.39.5 suite for analysis [99]. After assembly and barcode/primer removal, the sequences were processed for chimera removal using the *UCHIME* algorithm [100] and the SILVA reference set of 16S sequences (Release 128) [101]. Operational taxonomic unit (OTU) information was retrieved using a rarefied subset of 10,500 sequences per sample, randomly selected after multiple shuffling (10,000 ×) from the original dataset, and the *UCLUST* algorithm implemented in USEARCH v8.0.1623. OTUs were aligned using PyNAST [102] before tree building using FastTree [103] for diversity metrics based on phylogenetic distance. Common alpha diversity descriptors including the observed OTUs, Chao’s richness, Simpson’s evenness, dominance, phylogenetic distance, and Simpson’s reciprocal index were computed using *QIIME* v1.9.1 [104]. The information derived from the OTU-picking approach was also used to evaluate the β-diversity with the respective algorithms implemented in *QIIME* v1.9.1. The community structure was visualized by principal coordinate analysis (PCoA) from Bray–Curtis dissimilarity indexes retrieved from sample pairwise comparisons.

### Statistical Analysis

All animal data were analyzed using GraphPad Prism 7.0/8.0 (La Jolla, CA). Before the statistical analysis, the normality of the data was analyzed using different normality analysis tests (Anderson–Darling test, D’Agostino and Pearson test, Shapiro–Wilk test, Kolmogorov–Smirnov test). Statistical tests used for comparisons include one-way analysis of variance (ANOVA) with Tukey’s post hoc test for the binge (energy intake during the binge, chow binge, sucrose or water binge, and weight), neuropeptide, and receptor expression measures. Behavioral results were analyzed by either one-way ANOVA with post hoc Tukey’s test or by the Brown–Forsythe and Welch ANOVA test with post hoc Dunnett’s test. The circadian rhythms results were analyzed by two-way-ANOVA followed by post hoc Bonferroni or Tukey’s test. The results of microdialysis were analyzed by two-way-ANOVA followed by post hoc Bonferroni *or* Tukey’s test or by Kruskal–Wallis test followed by post hoc Dunn’s test. The immunohistochemistry data were analyzed by the Kruskal–Wallis test followed by the post hoc Dunn’s test. Data are presented as mean ± S.E.M.

Statistical analyses of gut microbiota data were carried out applying non-parametric methods including the Kruskal–Wallis and pairwise Wilcoxon rank sum tests (for unpaired samples) with Benjamini–Hochberg post hoc correction for multiple comparisons (alpha diversity descriptors). A linear discriminant analysis (LDA) was performed to compare the abundance of taxonomic signatures (OTUs, which are potential bacterial species) among different experimental groups. Differences were identified when OTUs had an LDA score ≥ 3.0. A permutation-based approach (PERMANOVA) was applied to evaluate changes in the microbiota structure based on the Bray–Curtis dissimilarity index. PCoA was carried out to visualize changes in microbiota composition among groups. Microbiota statistical tests and graphs were performed in R v3.6. Differences were considered statistically significant at p < 0.050.

## Supplementary Information

Below is the link to the electronic supplementary material.

Figure S1. B. *uniformis* modulates extracellular corticosterone in the nucleus accumbens but not in intestinal content in a rat model of food addiction. Microdialysis guide was implanted in the nucleus accumbens. Extracellular concentration of corticosterone (CORT) (A, B) was measured by microdialysis in freely moving rats and in intestinal content of the large intestine (C). Values are expressed in absolute value (nM) (A) and as the mean (nM) (B, C). Abbreviations: C, control group (n=9 in A, B and n=15 in C); IF, rats that fasted 12 h daily and received vehicle (n=10 in A, B and n=15 in C); IF B, rats that fasted 12 h and received a daily dose of 1×108 CFU B. *uniformis* (n=9 in A, B and n=15 in C). Two-way-ANOVA followed by post hoc Bonferroni was performed in the figure A and one-way ANOVA followed by post hoc Tukey´s or Kruskal-Wallis test post hoc Dunn´s was performed in the figures B, C. Statistically significant differences compared with the control group are indicated by an asterisk (*), different from IF group are indicated by (#), different from the control(c) group are indicated by (a), different from IF(c) group are indicated by (b), different from the control(c-p) group are indicated by (+), different from IF(c-p) group are indicated by (%). ** p<0.01, *** p<0.001, # p<0.05, ## p<0.01, ### p<0.001, a p<0.05, aaa p<0.001, bbb p<0.001, ++ p<0.01, %%% p<0.001. (PDF 99 KB)

Figure S2. B. *uniformis* does not modulate the expression of serotonergic and noradrenergic receptors or serotonin and noradrenaline concentrations in the hypothalamus in a rat food addiction model. Effect of B. *uniformis* on the relative expression of 5-HT2C (A) and 5-HT1B (B) and levels of 5-HT (C) in the hypothalamus (mM/g), relative expression of α-2B adrenergic receptor (D) in hypothalamus (relative units) and levels of noradrenaline (E) in the hypothalamus (mM/g). Abbreviations: C, control group (n=15); IF, rats that fasted 12 h and received vehicle (n=15); IF+B, rats that fasted 12 h and received a daily dose of 1×108 CFU B. *uniformis* (n=15). One-way ANOVA followed by post hoc Tukey´s test was performed in all the figures. Statistically significant differences compared with the control group are indicated by an asterisk (*). *** p<0.001. (PDF 89 KB)

Supplementary file3 (18.4 KB)

## Data Availability

All data are real and guarantee the validity of experimental results.
