# Supplementary figures and images for: Bacteroides uniformis CECT 7771 Modulates the Brain Reward Response to Reduce Binge Eating and Anxiety-Like Behavior in Rat
Source: Mol Neurobiol. 2021 Jul 6;58(10):4959–79. doi: 10.1007/s12035-021-02462-2 (PMC8497301; doi:10.1007/s12035-021-02462-2)

**A**

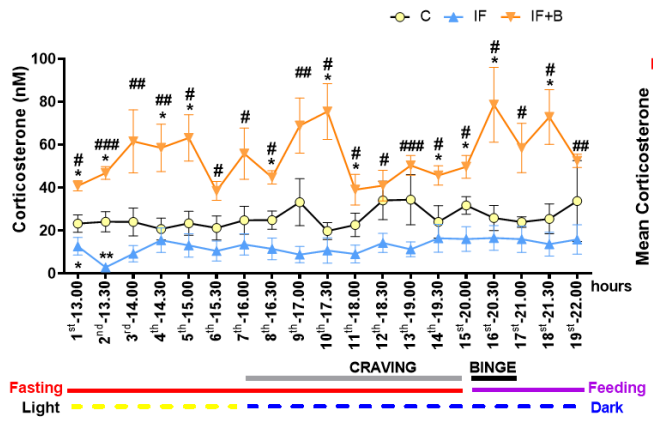

**B**

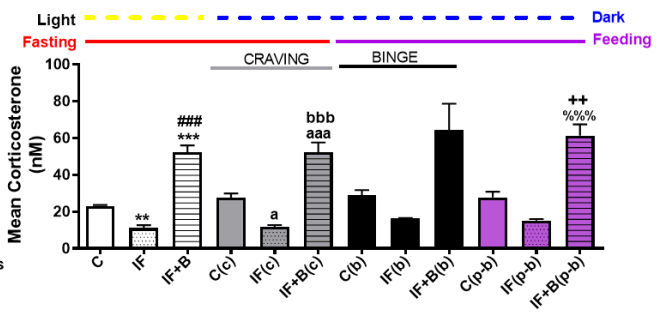

**C**

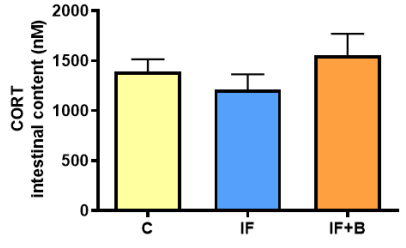

Supplement: Supplementary file 1 — Figure S1. B. uniformis modulates extracellular corticosterone in the nucleus accumbens but not in intestinal content in a rat model of food addiction. Microdialysis guide was implanted in the nucleus accumbens. Extracellular concentration of corticosterone (CORT) (A, B) was measured by microdialysis in freely moving rats and in intestinal content of the large intestine (C). Values are expressed in absolute value (nM) (A) and as the mean (nM) (B, C). Abbreviations: C, control group (n=9 in A, B and n=15 in C); IF, rats that fasted 12 h daily and received vehicle (n=10 in A, B and n=15 in C); IF B, rats that fasted 12 h and received a daily dose of 1×10^8^ CFU B. uniformis (n=9 in A, B and n=15 in C). Two-way-ANOVA followed by post hoc Bonferroni was performed in the figure A and one-way ANOVA followed by post hoc Tukey´s or Kruskal-Wallis test post hoc Dunn´s was performed in the figures B, C. Statistically significant differences compared with the control group are indicated by an asterisk (*), different from IF group are indicated by (#), different from the control(c) group are indicated by (a), different from IF(c) group are indicated by (b), different from the control(c-p) group are indicated by (+), different from IF(c-p) group are indicated by (%). ** p<0.01, *** p<0.001, # p<0.05, ## p<0.01, ### p<0.001, a p<0.05, aaa p<0.001, bbb p<0.001, ++ p<0.01, %%% p<0.001. (PDF 99 KB) [file 12035_2021_2462_MOESM1_ESM.pdf]

**A**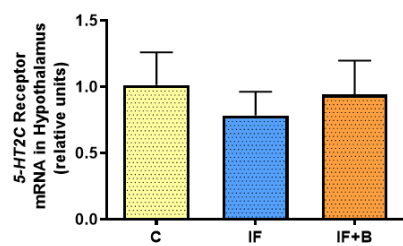**B**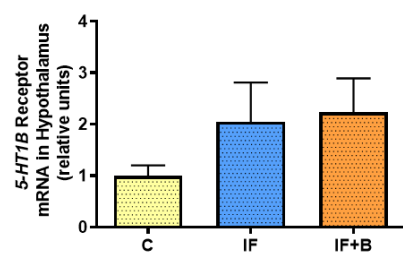**C**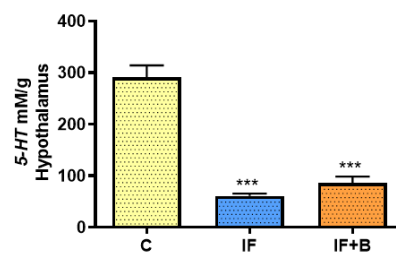**D**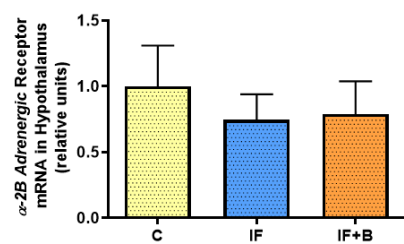**E**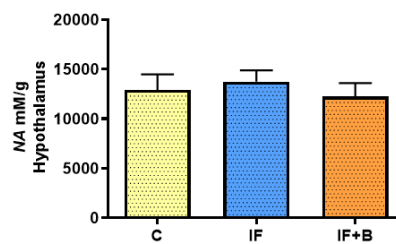

Supplement: Supplementary file 2 — Figure S2. B. uniformis does not modulate the expression of serotonergic and noradrenergic receptors or serotonin and noradrenaline concentrations in the hypothalamus in a rat food addiction model. Effect of B. uniformis on the relative expression of 5-HT2C (A) and 5-HT1B (B) and levels of 5-HT (C) in the hypothalamus (mM/g), relative expression of α-2B adrenergic receptor (D) in hypothalamus (relative units) and levels of noradrenaline (E) in the hypothalamus (mM/g). Abbreviations: C, control group (n=15); IF, rats that fasted 12 h and received vehicle (n=15); IF+B, rats that fasted 12 h and received a daily dose of 1×10^8^ CFU B. uniformis (n=15). One-way ANOVA followed by post hoc Tukey´s test was performed in all the figures. Statistically significant differences compared with the control group are indicated by an asterisk (*). *** p<0.001. (PDF 89 KB) [file 12035_2021_2462_MOESM2_ESM.pdf]
