# Supplementary material for: Bacteroides uniformis CECT 7771 Modulates the Brain Reward Response to Reduce Binge Eating and Anxiety-Like Behavior in Rat
Source: Mol Neurobiol. 2021 Jul 6;58(10):4959–79. doi: 10.1007/s12035-021-02462-2 (PMC8497301; doi:10.1007/s12035-021-02462-2)
Supplement: Supplementary file 3 — Supplementary file3 (18.4 KB) [file 12035_2021_2462_MOESM3_ESM.docx]

**Supplemental table 1. Reagents and tools.**

| **REAGENT or TOOLS** | **SOURCE** | **IDENTIFIER** |  |
| --- | --- | --- | --- |
| **Antibodies** | | |  |
| Anti-Dopamine Receptor D1 antibody | Abcam | ab40653 |  |
| Anti-Dopamine D2 Receptor | Abcam | ab191041 |  |
| **Bacterial and Virus Strains** | | |  |
| *Bacteroides uniformis* CECT 7771 | Yolanda Sanz laboratory (IATA-CSIC) | N/A |  |
| Wistar Kyoto male rats | Charles Rivers Laboratories | 008 |  |
| **Biological Samples** | | |  |
| Extracellular fluid from Nucleus Accumbens | Yolanda Sanz laboratory rats (IATA-CSIC) | N/A |  |
| Brain hippocampus | Yolanda Sanz laboratory rats (IATA-CSIC) | N/A |  |
| Brain hypothalamus | Yolanda Sanz laboratory rats (IATA-CSIC) | N/A |  |
| Intestinal content | Yolanda Sanz laboratory rats (IATA-CSIC) | N/A |  |
| Immunohistochemistry brain (prefrontal cortex) and small intestine slices | Instituto Valenciano de Patología (IVP) | N/A |  |
| **Chemicals, Peptides, and Recombinant Proteins** | | |  |
| Dopamine (DA) | Sigma | H8502-10G |  |
| Serotonin (5-HT) | Sigma | H9523-25MG |  |
| Noradrenaline (NA) | Sigma | 74480-100MG |  |
| Adrenaline (ADE) | Sigma | Y0000882 |  |
| Corticosterone (CORT) | Sigma | 27840-100MG |  |
| MediGel® Sucralose | Clear H2O | 74-02-5022 |  |
| **Experimental Models: Organisms/Strains** | | |  |
| *Bacteroides Uniformis* CECT 7771 (*B. uniformis*) | N/A | N/A |  |
| **Oligonucleotides** | | |  |
| Alpha 2B adrenergic receptor (α2B-adrenergic)  F: GCACCACAAAACCTGTTCCT  R: TTGTAGATGAGGGGCGGTAG | Isogen Life Science | N/A | |
| 5-HT2C receptor (5-HT2C)  F: GATGGTGGACGCTTGTTTCAATTCCCGGA  R: TTGACGGCGCAGGACGTAGATCGTTAAGA | Isogen Life Science | N/A | |
| 5-HT1B receptor (5-HT1B)  F: GCCGACGACTACATTTACCAGGACTCCAT  R: TGCCGGTCTTGTTGGGTGTCTGTTTCA | Isogen Life Science | N/A | |
| Neuropeptide Y (NPY)  F: GTGGACTGACCCTCGCTCTATC  R: ATGAGATTGATGTAGTGTCGCAGA | Isogen Life Science | N/A | |
| Agouti-related protein (AgRP)  F: CAGAGGTGCTAGATCCACAG  R: AGGTATTGAAGAAGCGGCAG | Isogen Life Science | N/A | |
| Pro-opiomelanocortin (POMC)  F: GAGGTTAAGGAGCAGTGACTAAGAG  R: GTAGCAGAATCTCGGCATCTTCC | Isogen Life Science | N/A | |
| Cocaine- and amphetamine-regulated transcript (CART)  F: GCTCAAGAGTAAACGCATTCC  R: AAGAATTGCAAGAAGTTCCTCG | Isogen Life Science | N/A | |
| Beta-2 microglobuline (β2m)  F: CGTGCTTGCCATTCAGAAAA  R: GAAGTTGGGCTTCCCATTCTC | Isogen Life Science | N/A | |
| **Software and Algorithms** | | |  |
| Prism 7.0 / 8.0 | GraphPad Software | https://www.graphpad.com/  scientific-software/prism/ |  |
| IMAGE J | National Institutes of Health | https://imagej.nih.gov/ij/ |  |
| SMART 2.0 | Panlab | https://www.panlab.com/en/products/smart-video-tracking-software-panlab |  |
| MOTHUR v1.39.5 | Mothur | <http://mothur.org/> |  |
| USEARCH v8.0.1623 | Usearch | https://www.drive5.com/usearch/ |  |
| QIIME v1.9.1 | Quiime | http://qiime.org/ |  |
| Illumina MiSeq platform | Illumina | <https://emea.illumina.com/products/by-system/miseq-products.html> |  |
